# Is There a Bias Towards Males in the Diagnosis of Autism? A Systematic Review and Meta-Analysis

**DOI:** 10.1007/s11065-023-09630-2

**Published:** 2024-01-29

**Authors:** Sara Cruz, Sabela Conde-Pumpido Zubizarreta, Ana Daniela Costa, Rita Araújo, Júlia Martinho, María Tubío-Fungueiriño, Adriana Sampaio, Raquel Cruz, Angel Carracedo, Montse Fernández-Prieto

**Affiliations:** 1 Psychology of Development Research Center, Lusiada University of Porto, Porto, Portugal; 2 Department of Psychology, School of Philosophy, Psychology & Language Sciences, University of Edinburgh, Edinburgh, UK; 3 Genomics and Bioinformatics Group, Center for Research in Molecular Medicine and Chronic Diseases (CiMUS), University of Santiago de Compostela (USC), Santiago de Compostela, Spain; 4 Psychological Neuroscience Laboratory (PNL), Research Center in Psychology (CIPsi), School of Psychology, University of Minho, Braga, Portugal; 5 Porto Nursing Higher School, Porto, Portugal; 6 Fundación Instituto de Investigación Sanitaria de Santiago de Compostela (FIDIS), Santiago de Compostela, Spain; 7 Genetics Group, GC05, Instituto de Investigación Sanitaria de Santiago (IDIS), Santiago de Compostela, Spain; 8 Grupo de Medicina Xenómica, U-711, Centro de Investigación en Red de Enfermedades Raras (CIBERER), University of Santiago de Compostela (USC), Santiago de Compostela, Spain; 9 Fundación Pública Galega de Medicina Xenómica, Servicio Galego de Saúde (SERGAS), Santiago de Compostela, Spain

**Keywords:** Meta-analysis, Systematic review, Autism, Gender differences, Camouflaging

## Abstract

Autism is more frequently diagnosed in males, with evidence suggesting that females are more likely to be misdiagnosed or underdiagnosed. Possibly, the male/female ratio imbalance relates to phenotypic and *camouflaging* differences between genders. Here, we performed a comprehensive approach to phenotypic and *camouflaging* research in autism addressed in two studies. First (Study 1 – Phenotypic Differences in Autism), we conducted a systematic review and meta-analysis of gender differences in autism phenotype. The electronic datasets Pubmed, Scopus, Web of Science, and PsychInfo were searched. We included 67 articles that compared females and males in autism core symptoms, and in cognitive, socioemotional, and behavioural phenotypes. Autistic males exhibited more severe symptoms and social interaction difficulties on standard clinical measures than females, who, in turn, exhibited more cognitive and behavioural difficulties. Considering the hypothesis of *camouflaging* possibly underlying these differences, we then conducted a meta-analysis of gender differences in camouflaging (Study 2 – *Camouflaging* Differences in Autism). The same datasets as the first study were searched. Ten studies were included. Females used more compensation and masking camouflage strategies than males. The results support the argument of a bias in clinical procedures towards males and the importance of considering a ‘female autism phenotype’—potentially involving *camouflaging*—in the diagnostic process.

**Supplementary Information:**

The online version contains supplementary material available at 10.1007/s11065-023-09630-2.

Autism spectrum disorder (ASD) is a neurodevelopmental condition characterised by persistent impairments in communication and social interaction, and restricted and repetitive patterns of behaviours, interests, or activities (American Psychiatric Association [APA], 2013). ASD prevalence has been increasing over the past decades (Hansen et al., 2015). Autistic^1^ individuals often display marked difficulties in everyday adaptive functioning, such as in peer relationship and social interactions (Harkins et al., 2022). An early diagnosis is thus critical to determining, implementing, and optimising early intervention programs, considering their positive impact on daily functioning and developmental outcomes (Kodak & Bergmann, 2020).

Autism is more prevalent in males than females, being diagnosed more often in boys than girls, with a male-to-female ratio of approximately 4:1 (Halladay et al., 2015; Lai et al., 2014). In the absence of intellectual disabilities, this ratio is even more pronounced, increasing to 10:1 (Fombonne, 2009; Rivet & Matson, 2011). Prominent gender differences in the prevalence and phenotype of autism may contribute to females being diagnosed later than males—commonly in adolescence or adulthood (Carter et al., 2007)—underdiagnosed, or not even receiving a diagnosis (Loomes et al., 2017). Autistic females often report significant mental health problems and poorer well-being, partly because of vulnerabilities associated with being undiagnosed (Bargiela et al., 2016).

Studies show that the clinical presentation of autism may differ between genders (Head et al., 2014; Van Wijngaarden-Cremers et al., 2014). Extensive research has explored possible differential phenotypic profiles between autistic females and males aimed at explaining this gender imbalance in the diagnosis (see Ferri et al., 2018, for a review). Literature points to phenotypic gender differences in multiple areas of functioning, such as in the core symptoms of autism, as well as in cognitive, socioemotional, and behavioural outcomes.

A recent meta-analysis documented that autistic females exhibit greater social interaction and social communication skills than autistic males, when these skills were measured with behavioural methods (e.g. play behaviour) (Wood-Downie, Wong, Kovshoff, Cortese et al. 2021; Wood-Downie, Wong, Kovshoff, Mandy et al. 2021). In parallel, when using standard clinical measures, such as the Autism Diagnostic Observation Schedule (ADOS), males exhibit more severe presentation of symptoms and greater communication impairments (Rea et al., 2023), as well as more pronounced social interaction difficulties (Mandy et al., 2012) and repetitive and stereotyped behaviours (Van Wijngaarden-Cremers et al., 2014) compared to females. Instead, autistic females are more likely to show more impaired functioning outcomes, such as worse intellectual performance (Frazier et al., 2014; Kreiser & White, 2013), adaptive functioning (Rubenstein et al., 2015), and greater internalising (Oswald et al., 2016) and externalising (Frazier et al., 2014; Guerrera et al., 2019) problems than males. However, it is important to note that findings on gender differences between autistic females and males are complex and may vary by some factors, such as intellectual or behavioural characteristics (Giambattista et al., 2021; Posserud et al., 2021).

It is possible that the current conceptualisation of autism leads to a diagnostic gender bias towards males (Haney, 2016). One explanation for this bias may be related to specifications of the diagnostic criteria of benchmark assessment instruments, such as the ADOS (Lord et al., 2012) and the Autism Diagnostic Interview-Revised (ADI-R; Rutter, Bailey et al., 2003; Rutter, Le Couteur et al., 2003). These instruments were validated using predominantly male individuals previously diagnosed with autism (Kirkovski et al., 2013; Kopp & Gillberg, 2011; Kreiser & White, 2013; Lai et al., 2015; Mattila et al., 2011), and thus lacking sex-based norms (McPartland et al., 2016). Therefore, conclusions drawn from primarily male samples may narrow the landscape of symptomatology to be assessed by not accurately capturing the female autism phenotype.

The hypothesis of a ‘specific female autism phenotype’ is supported by evidence showing that autistic females without intellectual impairments perform similarly to neurotypical females and higher than autistic males in social cognition tasks (e.g. visual attention to faces) (Harrop et al., 2020) and language abilities (e.g. use more appropriate language in social interactions) (Hiller et al., 2016), which contributes to their under-recognition. Furthermore, this hypothesis is also corroborated by higher levels of motivation for social relationships and fewer social impairments in autistic females, as well as lower levels of restricted and repetitive interests than males (Hull, Lai et al., 2020; Hull, Petrides et al., 2020). This apparent normal social functioning of autistic females seems associated with their ability to camouflage social behaviours to fit social environmental demands (Wood-Downie, Wong, Kovshoff, Cortese et al. 2021; Wood-Downie, Wong, Kovshoff, Mandy et al. 2021).

*Camouflaging* refers to the process by which autistic individuals, especially females, minimise the visibility of their autism to be considered more suitable and acceptable in social settings/interactions (Hull et al., 2017, 2019; Lai et al., 2017). According to Hull et al. (2017), *camouflaging* consists of three key coping strategies—*compensation* (i.e. actively performing behaviours aimed at overcoming social difficulties associated with autistic symptoms); *masking* (i.e. actively hiding autistic symptoms and/or difficulties); and *assimilation* (i.e. actively adopting observed behaviours and attitudes to blend in with others in social situations). In this paper, we will use the term ‘*camouflaging*’ and/or ‘camouflage’ to refer to these strategies.

Research in this area has shown that *camouflaging* is used mainly by autistic females to adapt their behaviour to different environments, especially as they feel greater pressure to fit into social situations (Hull, Lai et al., 2020; Hull, Petrides et al., 2020; Tubío-Fungueiriño et al., 2021). However, these behaviours come at a higher cost, as they have been associated with greater symptoms of anxiety and depression (Hull, Levy, et al., 2021), and are likely to cover specific autistic symptoms (Corbett et al., 2021).

Specific cognitive and other phenotypic traits may allow autistic females to camouflage autism-related social difficulties compared to autistic males, such as greater executive functioning abilities (e.g. cognitive flexibility; self-control skills), greater awareness of social norms (e.g. making eye contact), and of other’s cognitive and emotional states (e.g. Theory of Mind) (Hull, Petrides, et al., 2021; Livingston et al., 2019), as well as more social engagement and communication behaviours (Corbett et al., 2021). In addition, it may be that the ability to cover inappropriate social behaviours is shaped by socially constructed expectations directed at females regarding gender roles (Lai et al., 2015; Tubío-Fungueiriño et al., 2021). Autistic females are expected to display more pro-social behaviours and form closer relationships with others compared to autistic males (Hull et al., 2019).

## The Current Study 

Given the increasing prevalence and phenotypic differences between autistic females and males, as well as *camouflaging* in females, it is important to formally investigate these differences to possibly inform on their contribution to the imbalance of the male/female ratio in autism diagnosis. We address these questions in two studies. In ‘Study 1 – Phenotypic Differences in Autism’, we first investigated gender differences in the autism diagnosis, by unravelling the different diagnostic symptoms of ASD. To this end, we conducted a systematic review and meta-analysis of phenotypic differences between females and males in the core symptoms of autism (i.e. communication, social interaction and restricted interests and repetitive and stereotyped behaviour), and in cognitive (i.e. intellectual functioning), socioemotional (i.e. internalising problems) and behavioural (i.e. externalising behaviours) phenotypes. With this characterisation and considering that the clinical presentation of these symptoms may be affected by camouflage strategies, we then conducted ‘Study 2 – Camouflaging Differences in Autism’, a systematic review and meta-analysis of studies focusing on *camouflaging* in autistic females and males.

## Study 1 – Phenotypic Differences in Autism

Study 1 addresses gender differences in the core symptoms of autism, as well as in cognitive, socioemotional, and behavioural phenotypes. The protocol for conducting this investigation was registered in PROSPERO (CRD42021282480) and followed the 2020 PRISMA guidelines (Page et al., 2021).

## Method

### Literature Search

The electronic datasets Pubmed, Scopus, Web of Science, and PsychInfo were searched for empirical studies, published between 2013 (to reflect the latest autism diagnostic criteria as the DSM 5 was published this year) and December 2022. Studies were considered if (i) enrolled males and females with a diagnosis of autism or Asperger’s syndrome according to the DSM-IV-TR and/or DSM 5 (APA, 2013) diagnostic criteria; (ii) focused on sex/gender differences in the core symptoms of autism (i.e. communication, social interaction and restricted interests and repetitive and stereotyped behaviour); and (iii) in cognitive, socioemotional, and behavioural functioning outcomes. The following search terms were used: (‘asd’ OR ‘autis*’ OR ‘asperger’) AND ((‘sex difference’ OR ‘sex differences’) OR (‘sex characteristic’ OR ‘sex characteristics’) OR (‘gender difference’ OR ‘gender differences’)) AND ((‘social’ OR ‘social adaptation’ OR ‘interact*’) OR (‘behav*’ OR ‘stereotyp*’ OR ‘inflexib*’ OR ‘flexib*’ OR ‘ritual*’) OR (‘language’ OR ‘linguistic’ OR ‘communicat*’) OR (‘sensor*’ OR ‘sensory processing’)).

### Procedure

The initial database search resulted in 3555 articles, of which 1337 were duplicated. Hence, the title and abstract of 2218 articles were screened for the inclusion criteria by two independent researchers (RA and JM; *k* = 0.74). A third researcher (SC) acted as a consultant in case of conflict.

**Fig. 1:**
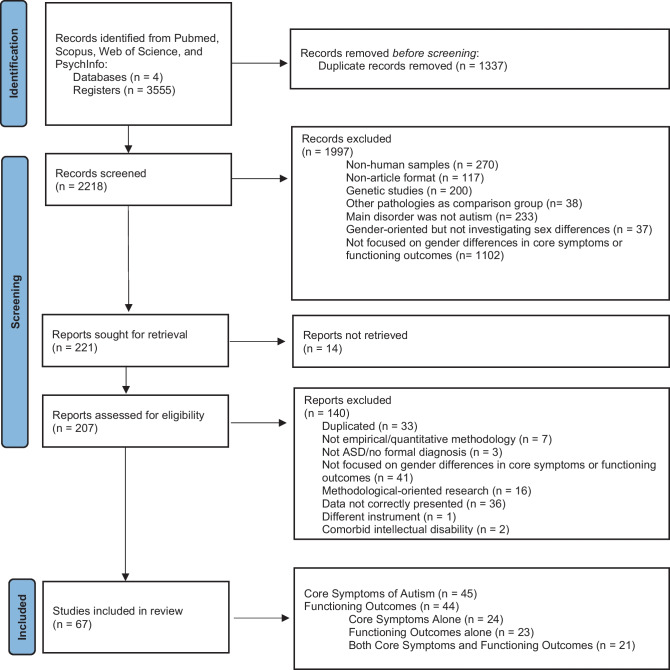
PRISMA flowchart depicting study selection procedures

Articles were excluded if (i) used non-human samples (*n* = 270); (ii) were not in article format (e.g. case reports, reviews, or meta-analysis) (*n* = 117); (iii) were genetic studies (*n* = 200); (iv) included other pathologies as a comparison group (*n* = 38); (v) the main pathology described was not autism or had a comorbid diagnosis (e.g. attention deficit/hyperactivity disorder [ADHD] or intellectual disability [ID]) (*n* = 233); (vi) were gender-oriented but not investigating gender/sex differences in autism (*n* = 37); and/or (vii) were not investigating gender differences in the core symptoms and functioning outcomes (i.e. cognition, socioemotional, and behaviour) in autism (*n* = 1102). As studies including autistic children with comorbid ID were excluded, studies including children with an intellectual coefficient (IQ) below 70 (i.e. IQ < 70) were not considered. Of the remaining 221 articles, 14 could not be retrieved. Thus, the screening resulted in 207 potentially relevant articles. The full text of these articles was retrieved and screened for inclusion criteria by two independent researchers (ADC and JM), and, in case of doubt or conflict, a consensus meeting was carried out with other researchers (SC and MFP). After a throughout and comprehensive examination of these articles, 140 were additionally excluded. This occurred because they were duplicated (*n* = 33), reported qualitative analysis (*n* = 7), did not provide information about the autism diagnosis (*n* = 3), did not focus on gender differences in the core symptoms or functioning outcomes (i.e. cognitive, socioemotional, and/or behaviour) (*n* = 41), were methodological-oriented research (e.g. assessing the discriminative characteristics of a questionnaire) (*n* = 16), did not present the data correctly (e.g. means and standard deviation values were not presented separately by gender, see Zhang et al., 2022, for an example) (*n* = 36), and used a different instruments to assess the dimensions under investigation (i.e. could not be included in the analysis due to the inability to compare results) (*n* = 1). Also, a study (Sturrock, Mardsen et al., 2020) was excluded as it reported the same results as another and earlier study (Sturrock, Yau et al., 2020, which was included in the analysis). Finally, two studies were removed as part of the study participants had comorbid ID (*n* = 2). Thus, 67 studies were included in the analysis. Figure 1 provides the flowchart of this selection procedure.

Some studies were included in more than one analysis, as they examined more than one area of interest for this review (e.g. a dimension of the core autistic symptoms, such as social interaction, and a functioning outcome, for example a cognitive functioning; see as an example Frazier et al., 2014). Overall, 45 articles compared females and males in the core symptoms of autism, whereas 44 compared genders regarding functioning outcomes. Of these, 24 addressed only the core symptoms, 23 only functioning outcomes, and 21 core symptoms and functioning outcomes combined (see Table 1 and 2 for detailed information about the included articles).

**Table 1:** Mean (M) values and standard deviations (SD) of age and measurement scores for the included articles addressing gender differences in the core symptoms of autism (i.e. total symptoms, communication, social interactions, and restricted interests and repetitive and stereotyped behaviour)

Article	Instrument	Age range in years (M; SD)	Males			Females			Total *N*
			***N***	**M**	**SD**	**N**	**M**	**SD**	
**Total symptoms**									
Baron–Cohen et al., 2015	AQ	18 + (M = 39.9; SD = 11.7)	178	36.93	8.64	217	34.71	10.97	395
Baron–Cohen et al., 2014	AQ	18–75 (M = 34.7; SD = 13.2)	357	34.8	9.1	454	32.9	11.5	811
Lai et al., 2017	AQ	18–49 (M = 27.5; SD = 7.5)	30	32.7	7.3	30	37.5	6.7	60
Rynkiewicz et al., 2016	AQ	5–10 (M = 8.15; SD = 1.8)	14	31.86	7.77	12	32.58	8.75	26
Schuck et al., 2019	AQ	18–55 (M = 28; SD = 6.9)	17	29.35	5.26	11	35.45	6.7	28
James et al., 2022	AQ	18–55 (M = 27.9; SD = 8.6)	12	29.3	5.5	11	35.5	6.7	23
Boorse et al., 2019	ADOS CSS	7–14 (M = 10.4; SD = 1.7)	41	6.71	2.37	21	6.38	2.64	62
	SCQ			20.27	7.01		20.29	5.21	
Cola et al., 2020	ADOS CSS	7–18 (M = 11.5; SD = 2.8)	25	7	1.8	15	6.6	2.29	40
	SCQ			17.56	7.88		17.79	7.39	
Craig et al., 2020	ADOS CSS	2–7 (M = 4.3; SD = 1.7)	62	6.21	1.19	52	5.32	1.7	114
Frazier et al., 2014	ADOS CSS	4–18 (M = 9.2; SD = 3.6)	2114	7.43	1.67	314	7.45	1.76	2428
	Vineland composite score			73.58	11.99		70.64	11.68	
Parish–Morris et al., 2017	ADOS CSS	6–17 (M = 9.96; SD = 2.05)	49	6.55	2.38	16	6.31	2.8	65
	SCQ			19.49	7.46		20.81	4.98	
	Vineland composite score			83.19	13.26		79.75	13.18	
Cola et al., 2022	ADOS CSS	6–15 (M = 10.4; SD = 1.9)	76	11.89	4.64	25	10.92	5.04	101
	SCQ			19.29	7.23		19.96	5.95	
Key, Jones et al., 2022; Key, Yan et al., 2022	ADOS CSS	10–16 (M = 12.8; SD = 1.9)	23	7.17	1.67	22	6.67	1.72	45
	SCQ			18.91	6.65		16.41	7.72	
Key, Jones et al., 2022; Key, Yan et al., 2022	ADOS CSS	10–16 (M = 12.9; SD = 1.6)	17	8	1.5	17	6.76	1.56	34
	SCQ			17.12	7.11		17.18	8.99	
Lawrence et al., 2022	ADOS CSS	8–17 (M = 13.6; SD = 2.7)	30	6.66	2.16	31	6.27	1.7	61
Lee et al., 2022	ADOS CSS	2–6 (M = 3.2; SD = 0.5)	189	7.44	1.8	93	7.37	1.7	282
Libster et al., 2022	ADOS CSS	6–15 (M = 10.4; SD = 1.8)	29	6.45	2.25	29	6.31	2.44	58
Neuhaus et al., 2022	ADOS CSS	8–17 (M = 12.5; SD = 2.9)	80	7.31	1.76	65	6.51	1.8	145
Osório et al., 2021	ADOS CSS	2–12.9 (M = 5.4; SD = 2.61)	138	7.26	1.81	26	6.35	1.85	164
Ross et al., 2022	ADOS CSS	4–17.9 (M = 8.9; SD = 3.6)	374	7.4	1.8	359	7.5	1.7	733
Song, Kim, et al., 2021	ADOS CSS	1.5–3.5 (M = 2.8; SD = 0.5)	207	6.45	1.56	54	6.92	1.84	261
	SCQ			14.62	5.87		14.64	5.87	
	Vineland composite score			69.63	12.76		69.45	16.21	
Waizbard–Bartov et al., 2022	ADOSCSS	3–11 (M = 3.1; SD = 0.5)	128	7	1.6	54	6.9	1.7	182
DaWalt et al., 2020	Vineland composite score	14–21 (M = 16.2; SD = 1.44)	471	75.54	0.8	76	77.28	2.26	547
	SCQ			20.73	0.41		20.86	0.99	
Goddard et al., 2014	SCQ	8–16 (M = 12.9; SD = 2.1)	12	26.5	5.92	12	20	5.15	24
Harrop et al., 2019	SCQ	6–10 (M = 8.9; SD = 1.1)	23	15	6.19	19	13.74	5.19	42
Nowell et al., 2019	SCQ	6–10 (M = 8.9; SD = 1.1)	27	14.92	5.94	27	13.92	5.02	54
Ros–Demarize et al., 2020	SCQ	4–6	51	16.94	6.12	18	18.17	6.65	69
Song et al., 2021	SCQ	8–16.7 (M = 11.6; SD = 2.5)	33	18.34	6.79	17	17.94	7.11	50
Mandic–Maravic et al., 2015	Vineland composite score	M = 6.73; SD = 4.33	83	73.58	11.99	25	70.64	11.68	108
White et al., 2017	Vineland composite score	7–18 (M = 12.4; SD = 2.6)	115	80.92	12.38	54	76.43	13.14	169
Reinhardt et al., 2015	Vineland composite score	M = 2.3; SD = 1	181	78.22	12.53	44	78.14	13.36	225
**Communication**									
Coffman et al., 2015	Vineland communication	8.3–13 (M = 10.2; SD = 1.7)	12	84.33	11.73	12	85.50	11.82	24
Frazier et al., 2014	Vineland communication	4–18 (M = 9.2; SD = 3.6)	2,114	77.59	14.58	314	74.3	13.71	2428
Mandic-Maravic et al., 2015	Vineland communication	M = 6.73; SD = 4.33	83	48.67	12.36	25	53.92	11.71	108
Parish-Morris et al., 2017	Vineland communication	6–17 (M = 9.96; SD = 2.05)	49	88.21	14.14	16	86.38	12.94	65
Reinhardt et al., 2015	Vineland communication	M = 2.3; SD = 1	181	82.69	16.82	44	79.43	17.61	225
White et al., 2017	Vineland communication	7–18 (M = 12.4; SD = 2.6)	130	84.87	13.60	57	84.40	15.57	187
Cola et al., 2022	Vineland communication	6–15 (M = 10.4; SD = 1.9)	76	86.92	13.52	25	87.4	12.21	101
Neuhaus et al., 2022	Vineland communication	8–17 (M = 12.5; SD = 2.9)	80	74.53	9.76	65	78.11	12.99	145
**Social interaction**									
Coffman et al., 2015	Vineland socialisation	8.3–13 (M = 10.2; SD = 1.7)	12	77.33	13.52	12	80.50	10.43	24
Frazier et al., 2014	Vineland socialisation	4–18 (M = 9.2; SD = 3.6)	2114	71.31	12.45	314	69.08	12.34	2428
	ADI–R social			9.33	3.73		9.22	3.41	
	ADOS social affect			11.01	3.96		11.55	4.24	
Mandic-Maravic et al., 2015	Vineland socialisation	M = 6.73; SD = 4.33	83	56.73	15.52	25	57.92	14.52	108
Parish–Morris et al., 2017	Vineland socialisation	6–17 (M = 9.96; SD = 2.05)	49	79.36	15.48	16	73.81	12.49	65
	ADOS social affect			6.29	2.38		6.31	2.63	
Reinhardt et al., 2015	Vineland socialisation	M = 2.3; SD = 1	181	78.02	11.9	44	78.55	13.14	225
White et al., 2017	Vineland socialisation	7–18 (M = 12.4; SD = 2.6)	130	77.98	13.35	56	74.41	12.96	186
Cola et al., 2022	Vineland socialisation	6–15 (M = 10.4; SD = 1.9)	76	77.22	14.92	25	73.6	11.92	101
	ADOS social affect			9.21	4.06		8.28	4.19	
	SRS total raw score			71.01	11.29		78.04	9.92	
Neuhaus et al., 2021	Vineland socialisation	8–17 (M = 12.3; SD = 2.9)	80	73.72	10.92	65	74.16	13.48	145
	ADOS social affect		81	7.28	1.89	61	6.61	1.79	
	SRS total–T score			90.38	28.02		78.22	11.55	
	SRS total raw score			72.6	11.18		95.71	27.33	
Boorse et al., 2019	ADOS social affect	7–14 (M = 10.4; SD = 1.7)	41	6.71	2.39	21	6.24	2.51	62
Cola et al., 2020	ADOS social affect	7–18 (M = 11.5; SD = 2.8)	25	7.36	1.66	15	6.53	2.20	40
Craig et al., 2020	ADOS social affect	2–7 (M = 4.3; SD = 1.7)	62	14.16	3.74	52	11.45	4.07	114
Lai et al., 2017	ADOS social affect	18–49 (M = 27.5; SD = 7.45)	30	8.50	5	30	4.30	3.60	60
Key, Jones et al., 2022; Key, Yan et al., 2022	ADOS social affect	10–16 (M = 12.9; SD = 1.6)	17	10.06	3.98	17	8.18	3.4	34
Libster et al., 2022	ADOS social affect	6–15 (M = 10.4; SD = 1.8)	29	6.69	2.17	29	6.34	2.32	58
Osório et al., 2021	ADOS social affect	2–12.9 (M = 5.4; SD = 2.61)	138	6.95	1.99	26	5.88	2.03	164
Song, Kim, et al., 2021	ADOS social affect	1.5–3.5 (M = 2.8; SD = 0.5)	207	7.33	1.72	54	7.64	2.08	261
	ADI–R social			16.21	5.25		14.1	4.71	
	SRS total–T score			63.83	10.42		64.67	11.09	
Waizbard–Bartov et al., 2022	ADOS social affect	3–11 (M = 3.1; SD = 0.5)	128	7.5	1.7	54	7.3	1.8	182
Supekar et al., 2022	ADI–R social	M = 13.3; SD = 6.2	126	17.7	7	552	17.9	6.5	678
Key, Jones et al., 2022; Key, Yan et al., 2022	SRS total–T score	10–16 (M = 12.8; SD = 1.9)	23	74.65	8.22	22	79.14	8.04	45
Lawrence et al., 2022	SRS total raw score	8–17 (M = 13.6; SD = 2.7)	30	97.43	31	31	98.27	32	61
	SRS total–T score			75.46	12.28		77.37	11.21	
Lee et al., 2022	SRS total–T score	2–6 (M = 3.2; SD = 0.5)	189	70.7	10.7	93	72.6	10.8	282
Milner et al., 2022	SRS total raw score	18.43–25.78 (M = 22.5)	34	77.68	24.8	41	100.85	25.25	751
O’Connor et al., 2022	SRS total raw score	9–16 (M = 11.6; SD = 1.3)	86	89.7	28.6	18	88.4	17.55	104
Ross et al., 2022	SRS total raw score	4–17.9 (M = 8.9; SD = 3.6)	374	94.8	26.1	359	99.9	27.3	733
Ko et al., 2022	SRS total–T score	11–16 (M = 13.4; 1.2)	22	75.45	8.31	10	74.4	11.52	32
**Restricted interests and repetitive and stereotyped behaviour**									
Boorse et al., 2019	ADOS RRB	7–14 (M = 10.4; SD = 1.7)	41	6.93	2.50	21	6.95	2.60	62
Cola et al., 2020	ADOS RRB	7–18 (M = 11.5; SD = 2.8)	25	6.44	2.14	15	7.27	1.75	40
Craig et al., 2020	ADOS RRB	2–7 (M = 4.3; SD = 1.7)	62	2.26	1.30	52	1.86	1.19	114
Frazier et al., 2014	ADOS RRB	4–18 (M = 9.2; SD = 3.6)	2114	3.96	2.05	314	4.01	2.21	2428
	RBS-R			27.1	17.29		26.86	16.93	
	ADI–R RRB			6.58	2.51		6.25	2.47	
Knutsen et al., 2019	ADOS RRB	2–12	512	7.60	1.80	512	7.50	2.10	1024
Lai et al., 2017	ADOS RRB	18–49 (M = 27.5; SD = 7.45)	30	8.5	5	30	4.3	3.6	60
McFayden et al., 2019	ADOS RRB	2–83 (M = 14.3; SD = 14.4	55	0.95	1.84	20	0.58	1.81	75
	RBS-R			4.96	3.41		3.23	2.78	
Parish–Morris et al., 2017	ADOS RRB	6–17 (M = 9.96; SD = 2.05)	49	7.27	2.32	16	6.50	3.14	65
Cola et al., 2022	ADOS RRB	6–15 (M = 10.4; SD = 1.9)	76	2.66	1.65	25	2.64	1.87	101
Key, Jones et al., 2022; Key, Yan et al., 2022	ADOS RRB	10–16 (M = 12.9; SD = 1.6)	17	4.18	1.74	17	3.24	1.39	34
Libster et al., 2022	ADOS RRB	6–15 (M = 10.4; SD = 1.8)	29	6.54	3.07	29	6.83	2.22	58
Neuhaus et al., 2021	ADOS RRB	8–17 (M = 12.3; SD = 2.9)	81	6.54	2.59	61	6.84	2.59	142
Osório et al., 2021	ADOS RRB	2–12.9 (M = 5.4; SD = 2.61)	138	7.7	1.88	26	7.27	2.44	164
Song, Kim, et al., 2021	ADOS RRB	1.5–3.5 (M = 2.8; SD = 0.5)	207	5.01	2.34	54	5.42	2.32	261
	ADI–R RRB			4.2	2.24		4.02	2.3	
Waizbard–Bartov et al., 2022	ADOS RRB	3–11 (M = 3.1; SD = 0.5)	128	8.3	1.6	54	8.1	1.6	182
Wang et al., 2017	ADI–R RRB	2–6.9 (M = 3.7; SD = 1.2)	836	4.55	2.06	228	3.59	1.87	1064
Supekar et al., 2022	ADI–R RRB	M = 13.3; SD = 6.2	126	5.6	2.6	552	5.3	2.7	678
Charman et al., 2017	RBS–R	6–30 (M = 16.7. SD = 5.8)	317	17.16	14.01	121	15.76	13.48	438
Harrop et al., 2018a	RBS–R	6–10 (M = 9; SD = 1.2)	25	28.80	16.91	26	34.38	23.03	51
Harrop et al., 2018b	RBS–R	6–10 (M = 8.6; SD = 1.6)	23	26.26	13.83	22	34.95	22.61	45
Nowell et al., 2019	RBS–R	6–17 (M = 9.96; SD = 2.05)	27	29.70	16.57	27	35.08	22.85	54

**Table 2:** Mean (M) values and standard deviations (SD) of age and measurement scores for the included articles addressing gender differences in functioning outcomes (i.e. cognitive, socioemotional, and behaviour)

Article	Measures	Age range in years (M; SD)	Males			Females			Total *N*
			***N***	**M**	**SD**	***N***	**M**	**SD**	
**Socioemotional and behavioural outcomes**									
Duvekot et al., 2017	CBCL externalising	2.5–10 (M = 6.7; SD = 2.3)	106	64.2	10.7	24	68.5	9.6	130
	CBCL internalising			63.3	9.9		70	8.1	
Frazier et al., 2014	CBCL externalising	4–18 (M = 9.2; SD = 3.6)	2114	56.37	10.62	314	58.04	10.10	2428
	CBCL internalising			60.37	9.47		60.15	9.76	
	Vineland DLS			76.88	13.81		73.51	13.53	
Pisula et al., 2017	CBCL externalising	11–18 (M = 13.8; SD = 2.1)	35	16.66	10.26	35	16.71	10.43	70
	CBCL internalising			20.74	11.42		24.34	11.37	
Postorino et al., 2015	CBCL externalising	2–5.4 (M = 3.55; SD = 0.9)	30	52.33	16.55	30	51.72	6.92	60
Prosperi et al., 2021	CBCL externalising	1.5–6.1 (M = 3.8; SD = 1.1)	107	54.06	10.46	107	52.87	9.56	214
	CBCL internalising			59.85	11.58		56.79	10.73	
Wiggins et al., 2021	CBCL internalising	2–5	1209	62.29	9.63	271	63.61	9.8	1480
Ross et al., 2022	CBCL internalising	4–17.9 (M = 8.9; SD = 3.6)	374	60.4	9.8	359	59.4	10.9	733
Mandic-Maravic et al., 2015	Vineland DLS	M = 6.73; SD = 4.33	83	62.71	18.94	25	70.8	18.05	108
	Vineland MS			73.67	15.29		79	14.73	
Reinhardt et al., 2015	Vineland DLS	M = 2.3; SD = 1	181	81.56	13.03	44	79.73	14.3	225
	Vineland MS			84.27	13.87		83.02	14.73	
White et al., 2017	Vineland DLS	7–18 (M = 12.4; SD = 2.6)	130	85.08	15.01	57	77.79	15.68	187
Neuhaus et al., 2021	Vineland DLS	8–17 (M = 12.3; SD = 2.9)	81	75.94	13.09	61	78.65	14.82	142
**Cognitive outcomes**									
Harrop et al., 2018a	DAS–II GCA	6–10 (M = 9; SD = 1.2)	25	115.23	31.69	26	96.75	32.48	51
Harrop et al., 2018b	DAS–II GCA	6–10 (M = 8.6; SD = 1.6)	23	120.51	26.36	22	98.55	34.61	45
Harrop et al., 2019	DAS–II GCA	6–10 (M = 8.9; SD = 1.1)	23	9.72	2.31	19	7.93	2.85	42
Parrish–Morris et al., 2017	DAS–II GCA	6–17 (M = 9.96; SD = 2.05)	49	106.00	14.00	16	104	13.00	65
Lawrence et al., 2022	DAS–II GCA	8–17 (M = 13.6; SD = 2.7)	30	105.2	15.82	31	100.77	22.43	61
Bitsika & Sharpley., 2019	WASI FSIQ	6–17 (M = 10.15; SD = 2.7)	32	95.8	13.7	32	99.9	12.8	64
Bitsika et al., 2018	WASI FSIQ	6–17 (M = 10.15; SD = 2.7)	51	97.9	12	51	98.2	13.1	102
Boorse et al., 2019	WASI FSIQ	7–14 (M = 10.4; SD = 1.7)	41	105.95	11.94	21	105.58	9.63	62
	WASI VIQ			105.46	11.7		108.95	11.35	
	WASI PIQ			106.54	12.89		108.14	11.93	
Coffman et al., 2015	WASI FSIQ	8.3–13 (M = 10.2; SD = 1.7)	12	98.82	19	12	100.17	20.06	24
Corbett et al., 2021	WASI FSIQ	10–16.1 (M = 12.8; SD = 1.9)	115	98.98	18.5	46	97.48	17.3	161
	WASI VIQ			97.98	18.5		100.5	16.2	
	WASI PIQ			99.77	20.2		95.96	19.3	
Cummings et al., 2020	WASI FSIQ	8–17 (M = 13.5; SD = 2.7)	37	106.76	13.6	16	100.63	19.2	53
Duvekot et al., 2017	WASI FSIQ	2.5–10 (M = 6.7; SD = 2.3)	106	95.3	17.4	24	99.9	18.9	130
	WASI VIQ		83	97.8	14.9	21	98.9	19.1	104
	WASI PIQ		95	97.7	17.9	21	102.9	13.8	116
	WISC FSIQ		101	95.3	17.4	22	99.9	18.9	123
	WISC VIQ		82	97.8	14.9	203	91.54	13.21	312
	WISC PIQ		95	97.7	17.9	21	102.9	13.8	116
Goddard et al., 2014	WASI FSIQ	8–16 (M = 12.9; SD = 2.1)	12	104.3	12.4	12	107.4	13.5	24
Lai et al., 2017	WASI FSIQ	18–49 (M = 27.5; SD = 7.45)	30	115.4	14.1	30	114.9	13.8	
	WASI VIQ			114.3	12.9		115.8	13.1	60
	WASI PIQ			113.3	15		110.4	16.7	
Lehnhardt et al., 2016	WAIS FSIQ	Not available	69	111.7	13.9	38	110.2	14.4	107
	WAIS VIQ			114.7	12.9		110	13	
	WAIS PIQ			106.2	15.9		108.3	15.6	
May et al., 2014	WASI FSIQ	7–12 (M = 9.9; SD = 1.9)	28	96.7	14.7	28	95.6	11.3	56
	WASI VIQ			98.5	15.3		99.25	13.3	
	WASI PIQ			104.6	15.8		95.7	13.1	
McFayden et al., 2019	WASI FSIQ	2–83 (M = 14.3; SD = 14.4	55	91.80	18.01	20	103.13	15.55	75
Sedgewick et al., 2019	WASI FSIQ	11–18 (M = 14.4; SD = 1.8)	26	104.92	16.11	27	99.15	16.47	53
	WASI VIQ			103.08	13.41		96.41	14.52	
	WASI PIQ			105.92	19.19		101.43	16.97	
Sedgewick et al., 2016	WASI FSIQ	12–16 (M = 13.5; SD = 1)	10	78.4	11.26	13	81.17	11.5	23
	WASI VIQ			79.5	12.14		77.77	11.28	
	WASI PIQ			81.2	16.09		84	15.38	
Song et al., 2021	WASI FSIQ	8–16.7 (M = 11.6; SD = 2.5)	33	105.27	13.03	17	109.76	11.99	60
	WASI VIQ			104.76	13.17		108.35	11.66	
	WASI PIQ			104.55	12.43		108.24	14.48	
Wilson et al., 2016	WASI FSIQ	18–75	163	99.4	17.6	41	92.4	20.2	204
	WASI VIQ		226	101.1	17.2	56	96.3	19.3	282
	WASI PIQ		223	95.2	17.9	56	92	19.1	279
Key, Jones et al., 2022; Key, Yan et al., 2022	WASI FSIQ	10–16 (M = 12.8; SD = 1.9)	23	101.87	18.33	22	100.05	17.55	45
	WASI VIQ			102.7	19.5		108.38	14.25	
	WASI PIQ			100.87	18.24		94.29	18.31	
Key, Jones et al., 2022; Key, Yan et al., 2022	WASI FSIQ	10–16 (M = 12.9; SD = 1.6)	17	99.94	18.23	17	99.29	17.16	34
	WASI VIQ			102	19.5		108.38	14.25	
	WASI PIQ			96.5	18.06		93.94	19	
Sturrock, Mardsen et al., 2020; Sturrock, Yau et al., 2020	WASI PIQ	8.1–11.1 (M = 10.1; SD = 0.8)	13	106.46	11.93	13	107.69	17.32	26
Conlon et al., 2019	WISC FSIQ	8–9 (M = 8.7; SD = 0.2)	671	88	15.99	203	87.46	16.86	874
	WISC VIQ			91.54	13.99		91.54	13.21	
	WISC PIQ			94	14.93		94.08	14.92	
Kauschke et al., 2016	WISC FSIQ	8–19 (12.5; 3.1)	11	99.73	13.36	11	98.46	14.02	22
	WISC VIQ			105	11.99		105.1	15.41	
	WISC PIQ			97.1	14.13		98.4	22.62	
Kumazaki et al., 2015	WISC FSIQ	5–9 (M = 7.5; SD = 1)	26	97.6	13.5	20	97.5	13.6	
	WISC VIQ			96.9	18.6		97.3	15.2	46
	WISC PIQ			98.5	11.3		98.3	12.7	
Mussey et al., 2017	WISC FSIQ	1.7–56.3 (M = 10.3; SD = 6.6)	566	85.98	21.8	113	85.59	22.1	679
	WISC VIQ			92.27	20.8		90.68	21	
	WISC PIQ			94.74	19.8		89.65	20.8	
Nasca et al., 2020	WISC FSIQ	6–12 (M = 9; SD = 1.8)	40	103.35	13.75	40	103.25	15.93	80
Rodgers et al., 2019	WISC FSIQ	6–12 (M = 8.9; SD = 1.7)	34	104.44	13.99	34	104.64	16.04	68
	WISC VIQ			104.1	14.15		104.03	16.09	
	WISC PIQ			103.84	16.53		104.74	16.4	
Kiep & Spek et al., 2017	WISC FSIQ	19–60 (M = 36.5; SD = 10)	99	109.62	12.44	40	107.86	12.14	139
	WISC VIQ			108.57	14.26		108.09	11.31	
Harrop et al., 2017	MSEL nonverbal age equivalent	2–5.9 (M = 3.8; SD = 0.6)	14	23.35	7.86	14	27.12	10.27	28
Harrop, Gulsrud et al., 2015; Harrop, Shire et al., 2015	MSEL nonverbal age equivalent	3–4 (M = 3.1; SD = 1.3)	29	30.2	6.49	29	32.29	11.86	58
Harrop, Gulsrud et al., 2015; Harrop, Shire et al., 2015	MSEL nonverbal age equivalent	1.8–4.5 (M = 3.4; SD = 0.7)	40	32.93	12.68	40	33.64	12.03	80
Reinhardt et al., 2015	MSEL nonverbal age equivalent	M = 2.3; SD = 1	234	26.07	9.09	54	25.64	8.85	288

### Data Selection and Extraction

The following information was extracted from the articles: (i) sample size, (ii) gender distribution, (iii) autism diagnosis information, and (iv) instruments used and scores for the dimensions investigated. Age was not considered as studies use numerous and different instruments and these are age-specific (see Table 1 and 2 for details on age). In total, the current study included 16,066 autistic individuals, of which 10,917 were males and 5149 females.

Different instruments were used across the studies to measure the core symptoms and functioning outcomes in autistic individuals. The following instruments were used to measure the core symptoms of autism: the ADOS (calibrated severity score—CSS; social affective—SA; and restricted and repetitive behaviours scale—RRB) (*n* = 21); the ADI-R (communication and restricted and repetitive behaviours scales) (*n* = 4); the Autism Spectrum Quotient (AQ; total score) (*n* = 6) (Baron-Cohen et al., 2001); the Social Communication Questionnaire (SCQ; total score) (*n* = 13) (Rutter, Bailey et al., 2003; Rutter, Le Couteur et al., 2003); the Repetitive Behaviour Scale Revised (RBS-R; total score) (*n* = 6) (Lam & Aman, 2007); the Vineland of Adaptive Behaviour Scales, Second Edition (Vineland-II; communication, socialisation and maladaptive behaviour scales, and the composite score) (*n* = 12) (Sparrow et al., 2005); and the Social Responsiveness Scale 2 (SRS-2; total raw score and total T score) (*n* = 10) (Constantino, 2013). Although the Vineland-II does not target autism-specific symptoms, we have included the communication, socialisation, and maladaptive behaviour scales in the core symptoms because they provide valuable clinical information that reflects the core symptoms of autism and on adaptive behaviour that may inform about the diagnosis of developmental disabilities (Dupuis et al., 2021; Milne et al., 2019). All instruments are parent report but the ADOS and the ADI-R are clinician screening reports.

Cognitive functioning was assessed using the full scale intelligence quotient (FSIQ), verbal intelligence quotient (VIQ) and performance intelligence quotient (PIQ) of the Weschler Abbreviated Scale of Intelligence (WASI; Wechsler, 1999) (*n* = 17) or the Wechsler Adult Intelligence Scale (WAIS; Wechsler, 2008) (*n* = 2) and of the Wechsler Intelligence Scale for Children (WISC; Wechsler, 2008) (*n* = 8), the General Conceptual Ability (GCA) of the Differential Ability Scales, Second Edition (DAS-II; Elliot et al., 2018) (*n* = 5), and the Non-verbal Age Equivalent scale of the Mullen Scales of Early Learning (MSEL; Mullen, 1995) (*n* = 4). The Child Behavioural Checklist (CBCL; Achenbach & Ruffle, 2000), a parent report questionnaire, was used to measure socioemotional difficulties (Internalising Problems scale) (*n* = 7) and behavioural problems (Externalising Problems scale) (*n* = 6). Additionally, the Vineland-II daily living skills scale (*n* = 5) was used to assess behavioural problems.

The quality and risk of bias were assessed independently using the Joanna Briggs Institute (JBI) critical appraisal checklist for analytic cross-sectional studies. Supplementary Material Table 1 provides information on the quality of the studies.

### Data Analysis

Meta-analyses of continuous outcome data were performed with *meta* R package. Analyses were computed separately for each instrument used to measure the multiple outcomes (e.g. the CSS scale of the ADOS, each subscale of the Vineland-II, the GCA of the DAS-II, or the Internalising and Externalising scales of the CBCL). A meta-analysis of the communication and restricted and repetitive behaviours scales of the ADI-R, the total score of the CBCL, and the Fine Motor, Visual Reception, and Receptive and Expressive Language scales of the MSEL was not computed as only two studies have used these measures and thus did not provide sufficient power to conduct the analysis (Ioannidis et al., 2008).

Standardised mean difference (SMD) was used as a summary measure for pooling studies. The SMD and 95% confident interval (CI) were calculated in R library using the default method (Hedges’ g method). A SMD above zero indicates that males scored higher than females and a SMD below zero indicates that females scored higher than males. Common and random effect estimates were obtained for inverse variance weighting meta-analyses. Heterogeneity was evaluated using the between-study variance (*t*^2^) and the I-squared (*I*^2^) statistics. For simplicity and considering the applicability of the results beyond the included studies, the random-effect results are discussed; for the dimensions with low heterogeneity (i.e. *p* > 0.05; *I*^2^ < 50%), the random-effects model is also considered.

## Results

**Fig. 2:**
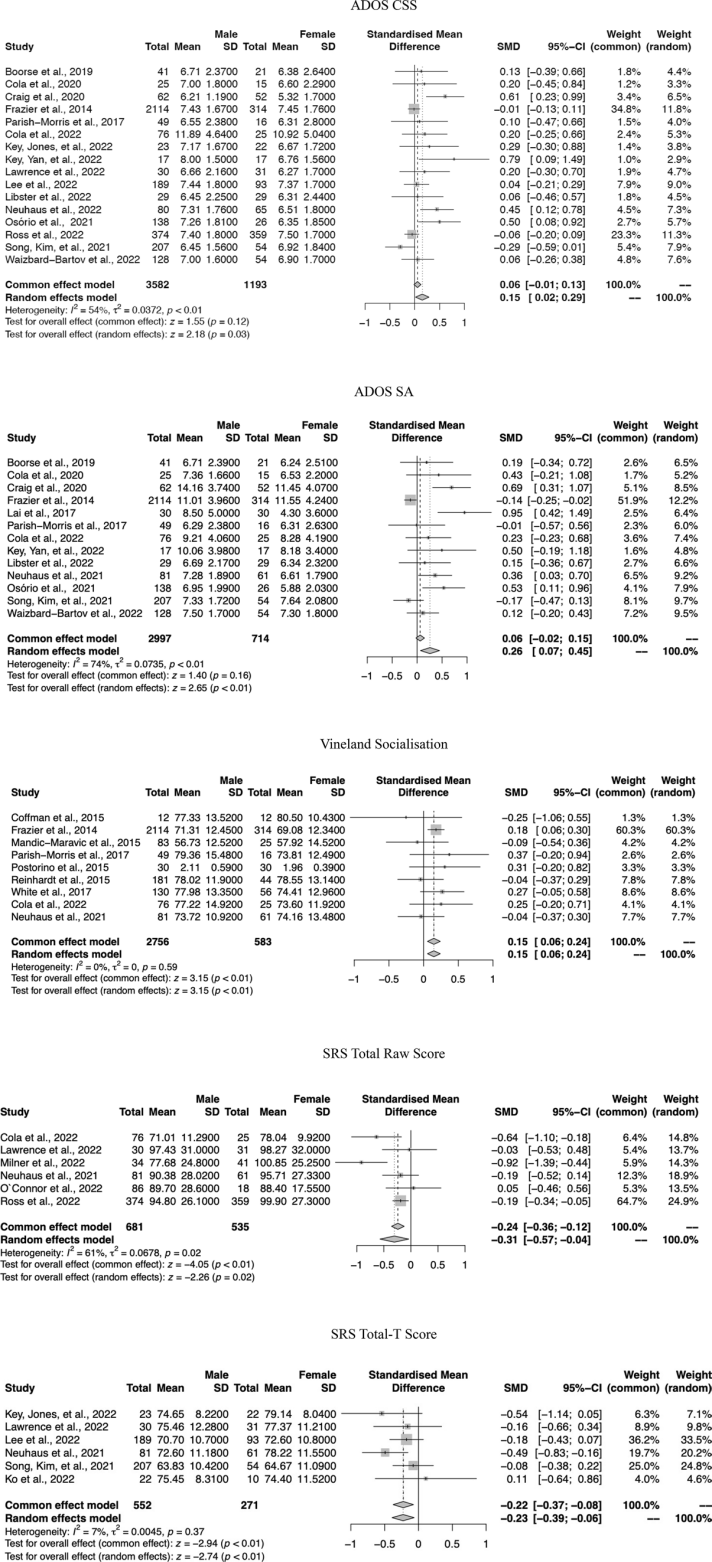
Forest plots of the gender differences in the autism core symptoms—severity of symptoms and social interaction difficulties

**Fig. 3:**
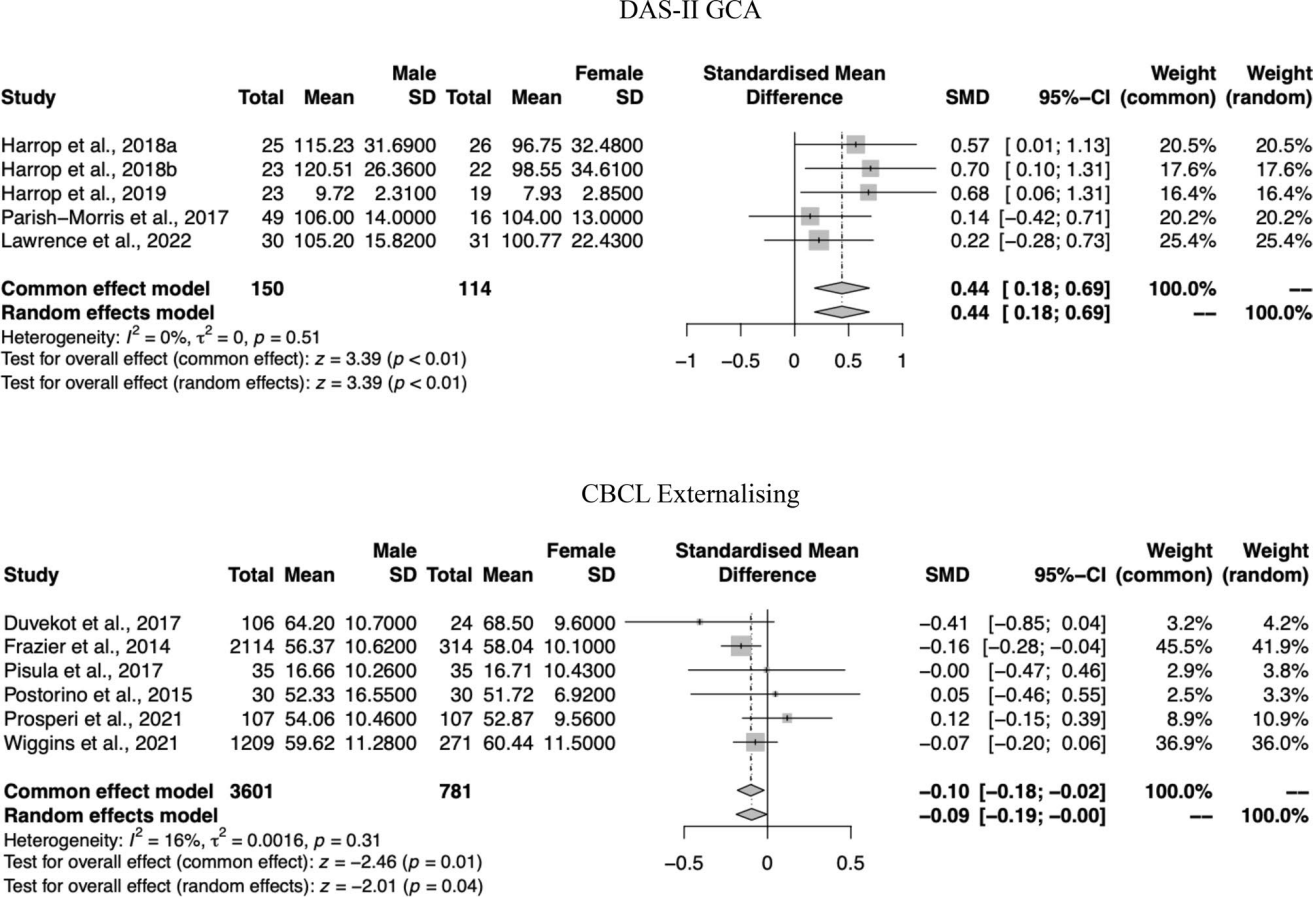
Forest plots of the gender differences in the cognitive and behavioural phenotypes

Figures 2 and 3 depict the forest plots of the gender differences in the autism core symptoms and in the cognitive, socioemotional, and behavioural phenotypes, respectively. The forest plots of the non-significant results are presented in Fig. 1 of the Supplementary Material.

### Autism Core Symptoms

#### Total Symptoms

The meta-analysis revealed significant gender differences in the CSS of the ADOS, SMD = 0.15, 95% CI = (0.02; 0.29), *z* = 2.18, *p* = 0.03. Autistic males presented worse severity scores compared to autistic females. No significant results were observed in the AQ, SMD = −0.29, 95% CI = (−0.75; 0.16), z = −1.27, *p* = 0.20; in the SCQ, SMD = −0.02, 95% CI = (−0.16; 0.12), *z* = −0.27, *p* = 0.79, or in the composite score of the Vineland-II, SMD = −0.15, 95% CI = (−0.65; 0.35), *z* = −0.58, *p* = 0.56.

#### Communication

There were no gender differences in the communication scale of the Vineland-II, SMD = −0.00, 95% CI = (−0.19; 0.19), *z* = −0.02, *p* = 0.99.

#### Social Interaction

The meta-analysis revealed significant gender differences in the SA subscale of the ADOS, SMD = 0.26, 95% CI = (0.07; 0.45), *z* = 2.65, *p* < 0.01. Autistic males presented more social interactive impairments than females. In addition, significant gender differences were found in the Socialisation scale of the Vineland-II, SMD = 0.15, 95% CI = (0.06; 0.24), *z* = 3.15, *p* < 0.01, in which autistic males presented more social interactive abilities than autistic females. The meta-analysis showed significant gender differences in the SRS-2 total raw score, SMD = −0.31, 95% CI = (−0.57; −0.04), *z* = −2.26, *p* = 0.02, and in the SRS-2 total-T score, SMD = −0.23, 95% CI = (−0.39; −0.06), *z* = −2.74, *p* < 0.01, in which autistic females presented more social interactive impairments than autistic males.

#### Restricted Interests and Repetitive and Stereotyped Behaviour

There were no significant gender differences in the RRB scale of the ADOS, SMD = 0.10, 95% CI = (−0.05; 0.24), *z* = 1.31, *p* = 0.19, or in the RBS-R total score, SMD = 0.02, 95% CI = (−0.08; 0.12), *z* = 0.37, *p* = 0.71.

### Cognitive, Socioemotional, and Behavioural Functioning Outcomes

The meta-analysis revealed gender differences in the GCA of the DAS-II, SMD = 0.44, 95% CI = (0.18; 0.69), *z* = 3.39, *p* < 0.01, in which males presented higher conceptual ability scores than females. No significant gender differences were observed in the WASI/WAIS FSIQ, SMD = −0.00, 95% CI = (−0.13; 0.12), *z* = −0.06, *p* = 0.95, VIQ, SMD = 0.01, 95% CI = (−0.15; 0.18), *z* = 0.17, *p* = 0.87, and PIQ, SMD = 0.09, 95% CI = (−0.05; 0.22), *z* = 1.25, *p* = 0.21, or for the WISC FSIQ, SMD = 0.02, 95% CI = (−0.09; 0.12), *z* = 0.36, *p* = 0.72, VIQ, SMD = 0.02, 95% CI = (−0.09; 0.13), *z* = 0.37, *p* = 0.71, and PIQ, SMD = 0.04, 95% CI = (−0.14; 0.21), *z* = 0.39, *p* = 0.70. Moreover, no gender differences were observed in the Non-Verbal Age Equivalent scale of the MSEL, SMD = −0.06, 95% CI = (−0.27; 0.15), *z* = −0.53, *p* = 0.60.

As for socioemotional and behavioural phenotypes, the meta-analysis showed gender differences in the Externalising Problems scale of the CBCL, SMD = −0.09, 95% CI (−0.19; −0.02), *z* = −2.01, *p* = 0.04, in which females present higher externalising problems scores than males. No statistically significant gender differences were observed in Internalising Problems scale of the CBCL, SMD = −0.05, 95% CI = (−0.25; 0.15), *z* = −0.50, *p* = 0.61, or in the Daily Living Skills of the Vineland, SMD = 0.08, 95% CI = (−0.22; 0.37), *z* = 0.51, *p* = 0.61.

## Discussion

This study examined phenotypic gender differences in autism core symptoms (i.e. communication, social interaction and restricted interests, and repetitive and stereotyped behaviour), and in cognitive (i.e. intellectual functioning), socioemotional (i.e. internalising problems), and behavioural (i.e. externalising behaviours) phenotypes. The meta-analysis revealed no gender differences in the domains of communication and restricted interests and repetitive and stereotyped behaviours. However, significant differences were observed between autistic females and males in the severity score of the ADOS and in the social interaction domain.

The results indicated that autistic females show a less severe presentation of autism symptoms than males when measured using the ADOS. This is consistent with previous studies and systematic reviews suggesting that males exhibit a more severe presentation of symptoms than females when assessed with clinical instruments (Waizbard-Bartov et al., 2022). Similarly, for the social interaction domain, the meta-analysis revealed that autistic males displayed increased social interaction difficulties compared to females in the ADOS, which is in accordance with other evidence (Mandy et al., 2012). However, when these dimensions were assessed with the SRS-2 or the Vineland-II scales, which are parent/caregiver or teacher (in the case of the SRS-2) reports, autistic females exhibited increased social interaction problems. This is in line with other research suggesting that autistic females are usually more impaired on parent-report measures of social functioning than males, despite the performance on standard diagnostic measures (Ratto et al., 2018). This may also be related to higher social expectations towards females, as they are expected to display more pro-social behaviours and establish closer social relationship with others (Tubío-Fungueiriño et al., 2021). When this is not the case, autistic females are likely to be perceived as having more difficulties in social interactions (Hull et al., 2019).

As for the functioning outcomes, the meta-analysis yielded gender differences in the cognitive and behavioural phenotypes. Particularly, autistic females presented more cognitive difficulties and externalising problems (e.g. defiance or aggressive behaviours) compared to autistic males. This is in line with other evidence suggesting that autistic females exhibit poorer intellectual functioning and more externalising behaviour difficulties, such as more irritability or self-injurious behaviours, than autistic males (Frazier et al., 2014). This seems to indicate that for females to receive a diagnosis of autism, they must present marked difficulties in overall functioning outcomes. For example, a study demonstrated that when females and males were rated similarly on diagnostic measures, females with higher IQs were less likely to meet the criteria for receiving a diagnosis of autism (Ratto et al., 2018). This is also in accordance with research indicating that autistic females need to exhibit more intellectual and behavioural problems to be captured by the current autism diagnostic criteria (Posserud et al., 2021).

In sum, the results seem to indicate that the clinical standard measures to assist an autism diagnosis are biased towards a male manifestation of ASD (Halladay et al., 2015; Loomes et al., 2017). Interestingly, even with comparable levels of symptom severity, females are less likely than males to receive a diagnosis of autism (Geelhand et al., 2019). It is possible that a female presentation of autism, potentially marked by differences in symptoms severity manifestation and social interaction skills compared to males, is not being captured by the current clinical procedures, which may constitute a barrier for females being properly diagnosed (Estrin et al., 2021). Symptoms and difficulties of autistic females may be expressed differently from the traditional, male-biased diagnostic criteria for autism, or may even express characteristics and/or behaviours that are not included in these criteria. In fact, one hypothesis that has gained increasing interest in the literature as underpinning the female autism phenotype is *camouflaging*, which appears to additionally support gender differences in the manifestation of autistic traits (Hull, Lai et al., 2020; Hull, Petrides et al., 2020). In addition, given the differences observed between clinician- and parent-reported measures, it is possible that females may be more motivated or able to camouflage during clinical assessments (i.e. more structured interactions), whereas a parent would be aware of difficulties in less structured settings (e.g. home). Although *camouflaging* had been attributed to autistic females, a comprehensive approach is needed to understand gender differences in the use of camouflage strategies/behaviours. To address this issue, we conducted a systematic review and meta-analysis comparing *camouflaging* between autistic females and males.

## Study 2 – Camouflaging Differences in Autism

Study 2 addresses gender differences in the use of camouflage strategies. This study also extends a previous systematic review conducted by our research team (Tubío-Fungueiriño et al., 2021) on *camouflaging* in autistic females.

## Method

### Literature Search

This study expands on a previous systematic review conducted by our research team (Tubío-Fungueiriño et al., 2021) on *camouflaging* in autistic females. In our previous research, we performed a literature search for empirical articles published between January 2009 and September 2019, which resulted in 13 studies that were included in that systematic review. Here, we conducted an additional search for articles published between October 2019 and October 2022. The same electronic databases described in study 1 and in Tubío-Fungueiriño et al. (2021) were searched for empirical studies in English. Studies were considered if (i) enrolled males and females with a diagnosis of autism or Asperger’s syndrome according to the DSM-IV-TR and/or DSM 5 diagnostic criteria (APA, 2013), and (ii) were focused on camouflaging, masking, compensation, assimilation, copy, or imitation behaviours of autistic symptoms in females. The following search terms were used: (‘autism’ OR ‘asd’ OR autis OR ‘asperger’) AND (‘gender’ OR ‘girls’ OR ‘woman’ OR ‘women’ OR ‘female*’ OR (sex AND difference)) AND (camoufla OR mask OR copy OR compensat OR imitat*).

### Procedure

The database search resulted in 1268 articles, of which 400 were duplicated. Thus, the title and abstract of 868 articles were screened for the inclusion criteria by two researchers (SCZ and MTF). Two other researchers (SC and MF) acted as consultants in case of any conflict.

Articles were excluded if (i) used non-human samples (*n* = 53); (ii) were not in article format (e.g. case reports, reviews, or meta-analysis) (*n* = 47); (iii) the main pathology described was not autism or Asperger (*n* = 366); and (iv) were not focused on the study of camouflaging with an autistic population (*n* = 369). The screening resulted in 33 potentially relevant articles that were retrieved and screened for the inclusion criteria. Of these, one article could not be retrieved. After examining the remaining 32 articles, 10 were further excluded either because they did not include information about the diagnosis (*n* = 6), consisted of same gender participants (*n* = 3), or did not examine camouflaging in autistic population (*n* = 1).

**Fig. 4:**
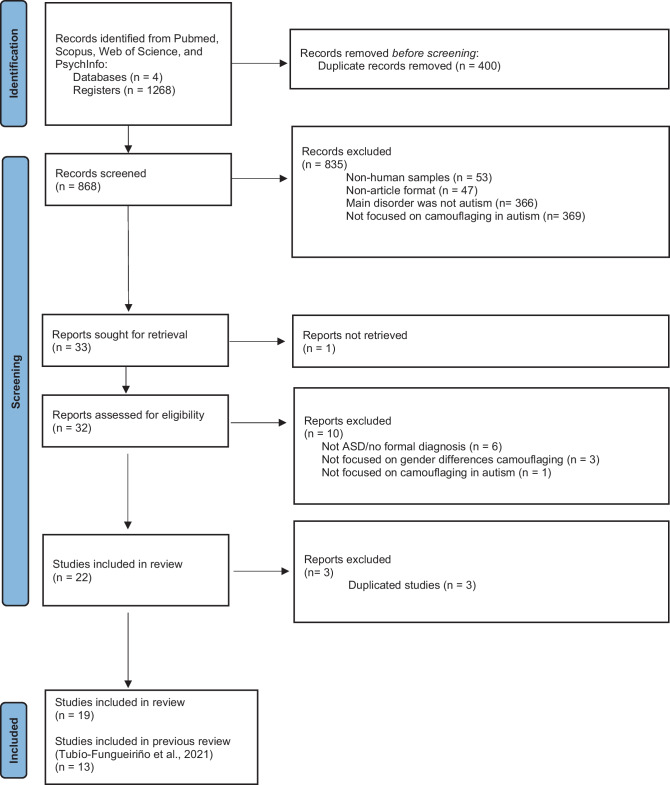
PRISMA flowchart depicting study selection procedures

Therefore, 22 studies were selected, and the full text was retrieved and screened for inclusion criteria. At this moment, three studies were excluded because they enrolled the same participants as described in other studies thus reporting the same results. We decided to include Cook et al. (2021) and Jorgenson et al. (2020) because they were conducted first and focused on camouflage behaviours in autistic individuals. These 19 studies were added to the 13 articles previously included in our systematic review (Tubío-Fungueiriño et al., 2021), resulting in 32 studies, whose full text was examined. Figure 4 depicts the flowchart of the selection procedures.

### Data Selection and Extraction

The following information was extracted from the articles: (i) sample size, (ii) gender distribution, (iii) autism diagnosis information, and (iv) instruments used and scores for measuring camouflaging behaviours.

Of the 32 studies, 15 measured *camouflaging* using The Camouflaging Autistic Traits Questionnaire (CAT-Q), six used the discrepancy method (i.e. capture and compare individuals’ scores on different measures), one used measures of social ability, and 10 conducted qualitative methods, such as observation (*n* = 3), or author-developed surveys (*n* = 7). However, due to the optimal statistical power to perform the meta-analysis, only the studies that used the CAT-Q were considered. Of the 15 studies, five were excluded because they did not report camouflaging score separated by gender.

Ten studies investigating *camouflaging* in autistic individuals were included in the meta-analysis (see Table 3 for detailed information about these studies). The quality and risk of bias of these were assessed using the JBI (Supplementary Material Table 2). Three studies were conducted with adolescents and seven with adults. Five studies reported gender differences considering only the total score of the CAT-Q, while six reported gender differences considering not only the total score, but also the scores of the additional three subscales—compensation, assimilation, and masking. Of the six studies that reported all the CAT-Q scores (the total and the three subscale), two were conducted with adolescents and four with adults. In addition, three studies enrolled non-binary autistic individuals, of which one presented only the CAT-Q total score and two the CAT-Q total and the subscales scores.

**Table 3:** Mean (M) values and standard deviations (SD) of age and CAT-Q total score and compensation, masking, and assimilation subscales scores for the included articles addressing camouflaging in autism

Article	CAT-Q	Age range in years (M; SD)	Males			Females			Total *N*
			***N***	**M**	**SD**	***N***	**M**	**SD**	
Belcher et al., 2022	Total score	18–40 (M = 25.6; SD = 14)	20	114.47	27.06	20	123.2	28.76	40
	Compensation			39.53	11.4		42.6	12.68	
	Masking			34.58	11.93		38.5	11.17	
	Assimilation			40.37	8.45		42.05	12.25	
Milner et al., 2022	Total	20–25 (M = 22.5)	34	97.35	22.27	42	108.76	24.33	76
	Compensation			35.26	11.2		42.04	11.86	
	Masking			43.02	13.25		44.46	13.2	
	Assimilation			39.01	9.08		44.2	9.59	
Hull, Lai et al., 2020; Hull, Petrides et al., 2020	Total	19–58 (M = 34.74; SD = 10.45)	108	109.64	26.50	182	124.35	23.27	290
	Compensation			36.81	12.14		41.85	11.11	
	Masking			32.9	10.57		37.87	10.54	
	Assimilation			39.93	11.26		44.63	7.82	
Hull, levy, et al., 2021; Hull, Petrides, et al., 2021	Total	13–18 (M = 14.5; SD = 1.7)	29	100.93	25.78	27	103.04	25.78	58
	Compensation			34.13	12.17		35.18	12.51	
	Masking			34.43	9.47		34.65	9.72	
	Assimilation			32.37	10.58		33.2	10.31	
Hull, Levy, et al., 2021	Total	18–75 (M = 41.9)	106	111.11	17.91	181	111.06	18.01	287
	Compensation			40.25	11.54		40.22	11.59	
	Masking			35.92	6.91		35.9	6.95	
	Assimilation			34.94	4.07		34.93	4.09	
Jorgenson et al., 2020	Total	13–18 (M = 15; SD = 1.7)	55	96.67	20.35	23	106.13	22.37	78
	Compensation			33.07	10.42		38.26	9.39	
	Masking			29.75	10.81		35.0	7.73	
	Assimilation			33.56	8.83		33.56	8.93	
Cage & Troxell-Whitman, 2019	Total score	18–66 (M = 33.6; SD = 11.5)	111	40.25	11.54	135	40.22	11.59	246
Cook et al., 2021	Total score	18–69 (M = 26.9; SD = 8.9)	6	35.92	6.91	8	35.9	6.95	14
Walsh et al., 2021	Total score	18–70 (M = 40.6; SD = 12.3)	21	120.21	30.34	24	107.00	23.29	45
Jedrzejewska & Dewey, 2022	Total score	13–19 (M = 14.1)	26	94.38	20.26	13	91.00	29.53	39

In total, the studies included in the meta-analysis enrolled 1172 participants, of whom 516 were males, 655 females, and 35 non-binaries. Of these, 998 were adults (592 males and 406 females) and 173 adolescents (110 males and 63 females).

### Data Analysis

Data analysis was conducted for the CAT-Q total score and for the subscales—compensation, assimilation, and masking. Gender comparisons were computed for adults and adolescents separately. A SMD above zero indicates that males display more camouflage behaviours, whereas a SMD below zero indicates that females present more camouflage behaviours. As in study 1, the random-effects results are discussed, regardless of the level of heterogeneity. Because no age differences were observed, results for overall effects are presented.

## Results

**Fig. 5:**
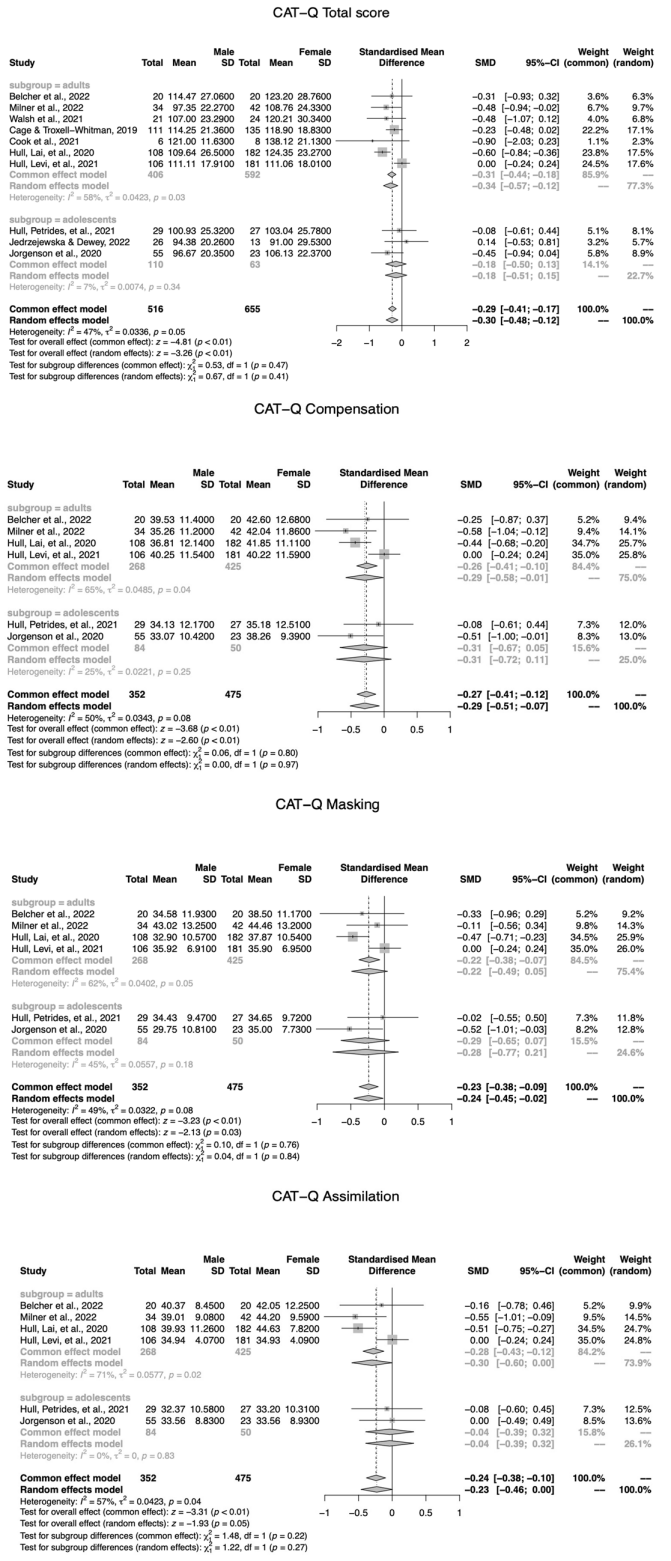
Forest plots of the gender differences in the CAT-Q total score and compensation, masking, and assimilation subscales

Figure 5 depicts the forest plots of the statistically significant gender differences in the CAT-Q total score and in the compensation, masking, and assimilation subscales.

The meta-analysis revealed that females scored higher than males in the total score of the CAT-Q, SMD = −0.30, 95% CI = (−0.48; −0.12), *z* = −3.26, *p* < 0.01. No significant gender differences were observed between females or males and non-binary participants. The meta-analysis revealed that females scored higher than males in the compensation, SMD = −0.29, 95% CI = (−0.51; −0.07), *z* = −2.60, *p* < 0.01, and masking, SMD = −0.24, 95% CI = (−0.45; −0.02), *z* = −2.13, *p* = 0.03, subscales. However, meta-analysis indicated no gender differences in the assimilation subscale, SMD = −0.23, 95% CI = (−0.46; 0.00), *z* = −1.93, *p* = 0.05.

## Discussion

This study investigated gender differences in *camouflaging* in autism. The results indicated that when using the CAT-Q, females exhibited higher total camouflaging scores than males. This supports the *camouflaging* hypothesis in females and is consistent with other literature documenting that autistic females *camouflage* more than autistic males, both in adolescence and adulthood (Beck et al., 2020; Dean et al., 2017; Hull, Lai et al., 2020; Hull, Petrides et al., 2020). We also observed that females utilise more masking and compensation strategies, but not assimilation, compared to males. Evidence is mixed regarding gender differences in the CAT-Q subscales, either reporting gender differences in masking and assimilation strategies, but not in compensation (Hull, Lai et al., 2020; Hull, Petrides et al., 2020), or in assimilation and compensation strategies, but not on masking (McQuaid et al., 2022). Similarly, evidence shows that non-binary autistic adults exhibit camouflage behaviours (Hull, Lai et al., 2020; Hull, Petrides et al., 2020; McQuaid et al., 2022), although they are not significantly different from autistic cisgender females or males, which was also observed in our results.

Studies point to the fact that autistic females appear to be more motivated to participate and engage in social interactions as they use different behavioural strategies to adapt to the demands of social environments (Wood-Downie, Wong, Kovshoff, Cortese et al. 2021; Wood-Downie, Wong, Kovshoff, Mandy et al. 2021). Our meta-analysis supports this evidence and further suggests that this appears to be achieved primarily through the utilisation of two camouflaging strategies—masking and compensation.

Masking is used to cover natural responses and adopt alternative behaviours to be accepted in social situations (Hull et al., 2019). Studies show that autistic females often mimic other people’s facial expressions (Cook et al., 2018), suppress repetitive behaviours (Wiskerke et al., 2018), or maintain appropriate eye contact (Lai et al., 2017) to conform to social group norms. Compensation refers to the use of strategies to overcome specific social difficulties associated with autistic symptoms (Hull et al., 2019). For example, some studies showed that females tend use more nonverbal communication or reciprocal communication on preferred topics (Corbett et al., 2021; Hiller et al., 2014), appear to pay attention to faces (Harrop et al., 2019), or recognise and infer other’s emotional states (Lai et al., 2017). In addition, one study demonstrated that autistic females who scored high on compensation strategies exhibited stronger social engagement and communication behaviours (Corbett et al., 2021).

Females seem to exhibit more socially appropriate behaviours than males, expressed mainly through masking and compensation strategies, which may prevent them from displaying the typical presentation of autism and therefore do not conform to the current diagnostic assessment criteria (McQuaid et al., 2022). This seems to be in line with the results of our first the meta-analysis, which indicated that when assessed with standard clinical measures, autistic females show less severe symptoms and social interactive difficulties. Furthermore, evidence suggests that high cognitive abilities (e.g. executive functions) are required to perform compensation and masking behaviours, as they need to self-monitor and inhibit innate behaviours (Hull, Levy, et al., 2021; Hull, Petrides, et al., 2021). This is equally in line with our first meta-analysis which that females need to present higher cognitive and behavioural problems to be diagnosed. It is possible that by masking and compensating for their autistic symptoms, females are more likely of being uncaptured by the current clinical criteria.

## General Discussion

This work has emphasised important gender differences in autism presentation, with females exhibiting lower symptom severity and impairments in social interaction. The underdiagnosis of autism in females may be due to a different expression of autistic symptoms compared to males, therefore not meeting current diagnostic criteria, as they perhaps manifest a specific female autism phenotype (Hull, Lai et al., 2020; Hull, Petrides et al., 2020).

Our results support the argument that current clinical diagnostic tools are biased towards males and that females’ autism presentation may be overlooked in the diagnostic process, especially if they do not present marked cognitive and/or behavioural difficulties. These findings have important implications for how clinicians measure autistic symptoms severity and social interaction difficulties, which certainly determines the assessment of behavioural symptoms in females. If diagnostically relevant behaviours, especially social-related behaviours, are being camouflaged, this may exacerbate the possibility of females being underdiagnosis or misdiagnosis (Cook et al., 2022). It is possible that autism is underdiagnosed in females because they camouflage their social difficulties and therefore conceal symptoms and do not meet current diagnostic criteria. This may reflect a different autistic profile between females and males and contribute to the imbalance in the diagnosis of autism.

Furthermore, research has shown that autistic females experience increased internalising and mental health problems because the try to ‘fit in’ socially by *camouflaging* their autistic traits (Beck et al., 2020). Other research has also shown that females who engage in camouflaging tend to deliberately inhibit ASD-related behaviours (i.e. externalising problems, such as repetitive behaviours) (Corbett et al., 2021). In sum, evidence suggests that for females to be diagnosed, they must present increased difficulties that, if camouflaged, are unlikely to be captured by current diagnostic procedures.

The lack of a better understanding of a possible female autism phenotype, potentially consisting of *camouflaging*, may hinder the accurate identification of autistic females (Allely, 2019). In accordance, recent *camouflaging*-oriented assessment tools, such as the CAT-Q, may be a useful measure to address this issue, capturing different forms of camouflaging strategies and, if integrated into clinical settings, aiding earlier and more accurate diagnoses, especially in females.

### Limitations and Future Directions

Although this work aimed to comprehensively understand gender differences in autism core symptoms, functioning outcomes, and *camouflaging*, synthesising evidence of this sort was challenging because studies use numerous and different assessment instruments. Because of this, and to not lose statistical power in the first study, the analysis was not computed controlling for participants’ age. This is a limitation. In particular, the significant results observed for the DAS (compared to other IQ measures) should be interpreted with caution. Developmental differences also contribute for the results (e.g. the heterogeneity in the trajectory of autistic symptoms across childhood, adolescence, and adulthood) and, thus, future studies should replicate this meta-analysis, controlling for age effects. In addition, the full range of IQ was not considered in this study as the presence of ID was an exclusion criterion. Other research should confirm our findings considering the full range of IQ. In the second study, despite including non-binary people in the analysis, they may not be in sufficient number to capture significant differences. It is important that future research continue to address possible differences in the expression of autistic traits in non-binaries. Importantly, we did not analyse the link between cognitive performance and the use of *camouflaging* strategies. Future studies should examine this relationship. Besides, the inclusion of only English language articles may not capture possible cultural variations that may be influencing the diagnosis, which should be account for in other investigations. Finally, future research should also include both quantitative and qualitative approaches to add to the comparison of camouflaging experiences across individuals.

## Conclusion

Epidemiological and clinical studies in autism have established a male predominance in autism prevalence, possible gender differences in autistic traits and a greater diagnostic difficulty for females. Perhaps females express their autistic traits differently, such as through *camouflaging*, and are therefore probably being underserved by the current conceptualisation and recognition of autism (Estrin et al., 2021). As a result, females may experience longer delays in clinical assessments and, consequently, are being underdiagnosed and/or misdiagnosed. It is thus important to improve and widespread the understanding and recognition of an autism presentation in females. Otherwise, autistic females may be at greater risk for marginalisation experiences, such as loss of personal, academic, and professional opportunities, loss of social support and understanding, and, consequently, at greater vulnerability to physical and mental health (e.g. anxiety and depression) problems.

## Supplementary Information

Below is the link to the electronic supplementary material.

Supplementary file1 (PDF 2384 KB)

Supplementary file2 (DOCX 30 KB)

## Data Availability

The data that support the findings of this study are available from the corresponding author upon request.

## Footnotes

1 To reflect the current feelings of members of the autism community, the term ‘autism’ will be used to refer to the clinical diagnosis of ASD, as ‘disorder’ is often associated with stigma and accentuates difficulties, ignoring possible strengths (Kenny et al., 2016). Equally, identity-first language (e.g., autistic person) instead of person-first language (e.g., individuals with autism) will be used to reflect the community wishes and recommendations for autism researchers (Bottema-Beutel et al., 2021).

## References

[CR1] Achenbach, T. M., & Ruffle, T. M. (2000). The Child Behavior Checklist and related forms for assessing behavioral/emotional problems and competencies. *Pediatrics in Review,**21*(8), 265–271. 10.1542/pir.21-8-265 10922023 10.1542/pir.21-8-265

[CR2] Allely, C. S. (2019). Understanding and recognising the female phenotype of autism spectrum disorder and the ‘camouflage’ hypothesis: A systematic PRISMA review. *Advances in Autism,**5*(1), 14–37. 10.1108/AIA-09-2018-0036

[CR3] American Psychiatric Association. (2013). *Diagnostic and Statistical manual of mental disorders* (5th ed.). 10.1176/appi.books.9780890425596

[CR4] Bargiela, S., Steward, R., & Mandy, W. (2016). The experiences of late-diagnosed women with autism spectrum conditions: An investigation of the female autism phenotype. *Journal of Autism and Developmental Disorders,**46*(10), 3281–3294. 10.1007/s10803-016-2872-8 27457364 10.1007/s10803-016-2872-8 PMC5040731

[CR5] Baron-Cohen, S., Cassidy, S., Auyeung, B., Allison, C., Achoukhi, M., Robertson, S., Pohl, A., & Lai, M. C. (2014). Attenuation of typical sex differences in 800 adults with autism vs. 3,900 controls. *PloS One*, *9*(7), e102251. 10.1371/journal.pone.0102251 10.1371/journal.pone.0102251 PMC4100876 25029203

[CR6] Baron-Cohen, S., Bowen, D. C., Holt, R. J., Allison, C., Auyeung, B., Lombardo, M. V., Smith, P., & Lai, M. C. (2015). The “reading the mind in the eyes” test: Complete absence of typical sex difference in ~400 men and women with autism. *PLoS ONE,**10*(8), e0136521. 10.1371/journal.pone.0136521 26313946 10.1371/journal.pone.0136521 PMC4552377

[CR7] Baron-Cohen, S., Wheelwright, S., Skinner, R., Martin, J., & Clubley, E. (2001). The autism-spectrum quotient (AQ): Evidence from Asperger syndrome/high-functioning autism, males and females, scientists and mathematicians. *Journal of Autism and Developmental Disorders,**31*(1), 5–17. 10.1023/a:1005653411471 11439754 10.1023/a:1005653411471

[CR8] Beck, J. S., Lundwall, R. A., Gabrielsen, T., Cox, J. C., & South, M. (2020). Looking good but feeling bad: “Camouflaging” behaviors and mental health in women with autistic traits. *Autism,**24*(4), 809–821. 10.1177/1362361320912147 32429817 10.1177/1362361320912147

[CR9] Belcher, H. L., Morein-Zamir, S., Mandy, W., & Ford, R. M. (2022). Camouflaging intent, First impressions, and age of ASC diagnosis in autistic men and women. *Journal of Autism and Developmental Disorders,**52*(8), 3413–3426. 10.1007/s10803-021-05221-3 34342806 10.1007/s10803-021-05221-3 PMC9296412

[CR10] Bitsika, V., & Sharpley, C. F. (2019). Effects of diagnostic severity upon sex differences in behavioural profiles of young males and females with autism spectrum disorder. *Journal of Autism and Developmental Disorders,**49*(11), 4429–4440. 10.1007/s10803-019-04159-x 31377944 10.1007/s10803-019-04159-x

[CR11] Bitsika, V., Sharpley, C. F., & Mills, R. (2018). Sex differences in sensory features between boys and girls with autism spectrum disorder. *Research in Autism Spectrum Disorders,**51*, 49–55. 10.1016/j.rasd.2018.04.002

[CR12] Boorse, J., Cola, M., Plate, S., Yankowitz, L., Pandey, J., Schultz, R. T., & Parish-Morris, J. (2019). Linguistic markers of autism in girls: evidence of a “blended phenotype” during storytelling. *Molecular Autism*, *10*(1). 10.1186/s13229-019-0268-2 10.1186/s13229-019-0268-2 PMC6436231 30962869

[CR13] Bottema-Beutel, K., Kapp, S. K., Lester, J. N., Sasson, N. J., & Hand, B. N. (2021). Avoiding ableist 400 language: Suggestions for autism researchers. *Autism in Adulthood,**3*(1), 18–29. 10.1089/aut.2020.0014 36601265 10.1089/aut.2020.0014 PMC8992888

[CR14] Cage, E., & Troxell-Whitman, Z. (2019). Understanding the reasons, contexts and costs of camouflaging for autistic adults. *Journal of Autism and Developmental Disorders,**49*(5), 1899–1911. 10.1007/s10803-018-03878-x 30627892 10.1007/s10803-018-03878-x PMC6483965

[CR15] Carter, A. S., Black, D. O., Tewani, S., Connolly, C. E., Kadlec, M. B., & Tager-Flusberg, H. (2007). Sex differences in toddlers with autism spectrum disorders. *Journal of Autism and Developmental Disorders,**37*(1), 86–97. 10.1007/s10803-006-0331-7 17216333 10.1007/s10803-006-0331-7

[CR16] Charman, T., Loth, E., Tillmann, J., Crawley, D., Wooldridge, C., Goyard, D., Ahmad, J., Auyeung, B., Ambrosino, S., Banaschewski, T., Baron-Cohen, S., Baumeister, S., Beckmann, C., Bölte, S., Bourgeron, T., Bours, C., Brammer, M., Brandeis, D., Brogna, C., Buitelaar, J. K. (2017). The EU-AIMS Longitudinal European Autism Project (LEAP): Clinical characterisation. *Molecular Autism*, *8*(1). 10.1186/s13229-017-0145-9 10.1186/s13229-017-0145-9 PMC5481972 28649313

[CR17] Coffman, M. C., Anderson, L. C., Naples, A. J., & Mcpartland, J. C. (2015). Sex differences in social perception in children with ASD. *Journal of Autism and Developmental Disorders,**45*(2), 589–599. 10.1007/s10803-013-2006-5 24293083 10.1007/s10803-013-2006-5 PMC4039621

[CR18] Cola, M. L., Plate, S., Yankowitz, L., Petrulla, V., Bateman, L., Zampella, C. J., De Marchena, A., Pandey, J., Schultz, R. T., & Parish-Morris, J. (2020). Sex differences in the first impressions made by girls and boys with autism. *Molecular Autism*, *11*(1). 10.1186/s13229-020-00336-3 10.1186/s13229-020-00336-3 PMC7298946 32546266

[CR19] Cola, M., Yankowitz, L. D., Tena, K., Russell, A., Bateman, L., Knox, A., Plate, S., Cubit, L. S., Zampella, C. J., Pandey, J., Schultz, R. T., & Parish-Morris, J. (2022). Friend matters: Sex differences in social language during autism diagnostic interviews. *Molecular Autism*, *13*(1). 10.1186/s13229-021-00483-1 10.1186/s13229-021-00483-1 PMC8751321 35012645

[CR20] Conlon, O., Volden, J., Smith, I. M., Duku, E., Zwaigenbaum, L., Waddell, C., Szatmari, P., Mirenda, P., Vaillancourt, T., Bennett, T., Georgiades, S., Elsabbagh, M., & Ungar, W. J. (2019). Gender differences in pragmatic communication in school-aged children with autism spectrum disorder (ASD). *Journal of Autism and Developmental Disorders,**49*(5), 1937–1948. 10.1007/s10803-018-03873-2 30627893 10.1007/s10803-018-03873-2

[CR21] Constantino, J. N. (2013). Social responsiveness scale. In F.R. Volkmar (Ed.), *Encyclopedia of Autism Spectrum Disorders* (pp 4457–4467). Springer. 10.1007/978-1-4419-1698-3_296

[CR22] Cook, A., Ogden, J., & Winstone, N. (2018). Friendship motivations, challenges and the role of masking for girls with autism in contrasting school settings. *European Journal of Special Needs Education,**33*(3), 302–315. 10.1080/08856257.2017.1312797

[CR23] Cook, J., Crane, L., Bourne, L., Hull, L., & Mandy, W. (2021). Camouflaging in an everyday social context: An interpersonal recall study. *Autism,**25*(5), 1444–1456. 10.1177/1362361321992641 33607921 10.1177/1362361321992641 PMC8264642

[CR24] Cook, J., Crane, L., Hull, L., Bourne, L., & Mandy, W. (2022). Self-reported camouflaging behaviours used by autistic adults during everyday social interactions. *Autism,**26*(2), 406–421. 10.1177/13623613211026754 34180249 10.1177/13623613211026754 PMC8814950

[CR25] Corbett, B. A., Schwartzman, J. M., Libsack, E. J., Muscatello, R. A., Lerner, M. D., Simmons, G. L., & White, S. W. (2021). Camouflaging in autism: Examining sex-based and compensatory models in social cognition and communication. *Autism Research,**14*(1), 127–142. 10.1002/aur.2440 33220170 10.1002/aur.2440 PMC7986572

[CR26] Craig, F., Crippa, A., De Giacomo, A., Ruggiero, M., Rizzato, V., Lorenzo, A., Fanizza, I., Margari, L., & Trabacca, A. (2020). Differences in developmental functioning profiles between male and female preschoolers children with autism spectrum disorder. *Autism Research,**13*(9), 1537–1547. 10.1002/aur.2305 32282130 10.1002/aur.2305

[CR27] Cummings, K. K., Lawrence, K. E., Hernandez, L. M., Wood, E. T., Bookheimer, S. Y., Dapretto, M., & Green, S. A. (2020). Sex differences in salience network connectivity and its relationship to sensory over-responsivity in youth with autism spectrum disorder. *Autism Research,**13*(9), 1489–1500. 10.1002/aur.2351 32860348 10.1002/aur.2351 PMC8351910

[CR28] DaWalt, L. S., Taylor, J. L., Bishop, S., Hall, L. J., Steinbrenner, J. D., Kraemer, B., Hume, K. A., & Odom, S. L. (2020). Sex differences in social participation of high school students with autism spectrum disorder. *Autism Research,**13*(12), 2155–2163. 10.1002/aur.2348 32881417 10.1002/aur.2348 PMC7749043

[CR29] Dean, M., Harwood, R., & Kasari, C. (2017). The art of camouflage: Gender differences in the social behaviors of girls and boys with autism spectrum disorder. *Autism,**21*(6), 678–689. 10.1177/1362361316671845 27899709 10.1177/1362361316671845

[CR30] Dupuis, A., Moon, M. J., Brian, J., Georgiades, S., Levy, T., Anagnostou, E., Nicolson, R., Schachar, R., & Crosbie, J. (2021). Concurrent validity of the ABAS-II questionnaire with the Vineland II interview for adaptive behavior in a pediatric ASD sample: High correspondence despite systematically lower scores. *Journal of Autism and Developmental Disorders,**51*(5), 1417–1427. 10.1007/s10803-020-04597-y 32776267 10.1007/s10803-020-04597-y

[CR31] Duvekot, J., van der Ende, J., Verhulst, F. C., Slappendel, G., van Daalen, E., Maras, A., & Greaves-Lord, K. (2017). Factors influencing the probability of a diagnosis of autism spectrum disorder in girls versus boys. *Autism,**21*(6), 646–658. 10.1177/1362361316672178 27940569 10.1177/1362361316672178

[CR32] Elliott, C. D., Salerno, J. D., Dumont, R., & Willis, J. O. (2018). The differential ability scales—Second edition. In D. P. Flanagan & E. M. McDonough (Eds.), *Contemporary intellectual assessment: Theories, tests, and issues* (pp. 360–382). The Guilford Press.

[CR33] Estrin, G. L., Milner, V., Spain, D., Happé, F., & Colvert, E. (2021). Barriers to autism spectrum disorder diagnosis for young women and girls: A systematic review. *Review Journal of Autism and Developmental Disorders,**8*(4), 454–470. 10.1007/s40489-020-00225-8 34868805 10.1007/s40489-020-00225-8 PMC8604819

[CR34] Ferri, S.L., Abel, T., & Brodkin, E.S. (2018). Sex differences in autism spectrum disorder: A review. *Current Psychiatry Reports, 20*(2). 10.1007/s11920-018-0874-2 10.1007/s11920-018-0874-2 PMC6477922 29504047

[CR35] Fombonne, E. (2009). Epidemiology of pervasive developmental disorders. *Pediatric Research,**65*(6), 591–598. 10.1203/pdr.0b013e31819e7203 19218885 10.1203/PDR.0b013e31819e7203

[CR36] Frazier, T. W., Georgiades, S., Bishop, S. L., & Hardan, A. Y. (2014). Behavioral and cognitive characteristics of females and males with autism in the Simons Simplex Collection. *Journal of the American Academy of Child and Adolescent Psychiatry,**53*(3), 329–340. 10.1016/j.jaac.2013.12.004 24565360 10.1016/j.jaac.2013.12.004 PMC3935179

[CR37] Geelhand, P., Bernard, P., Klein, O., van Tiel, B., & Kissine, M. (2019). The role of gender in the perception of autism symptom severity and future behavioral development. *Molecular Autism,**10*(1), 16. 10.1186/s13229-019-0266-4 30976383 10.1186/s13229-019-0266-4 PMC6439965

[CR38] Giambattista, C., Ventura, P., Trerotoli, P., Margari, F., & Margari, L. (2021). Sex differences in autism spectrum disorder: Focus on high functioning children and adolescents. *Frontiers in Psychiatry,**12*(1–13), 539835. 10.3389/fpsyt.2021.539835 34305658 10.3389/fpsyt.2021.539835 PMC8298903

[CR39] Goddard, L., Dritschel, B., & Howlin, P. (2014). A preliminary study of gender differences in autobiographical memory in children with an autism spectrum disorder. *Journal of Autism and Developmental Disorders,**44*(9), 2087–2095. 10.1007/s10803-014-2109-7 24777286 10.1007/s10803-014-2109-7

[CR40] Guerrera, S., Menghini, D., Napoli, E., Di Vara, S., Valeri, G., & Vicari, S. (2019). Assessment of psychopathological comorbidities in children and adolescents with autism spectrum disorder using the child behavior checklist. *Frontiers in Psychiatry*, *10*. 10.3389/fpsyt.2019.00535 10.3389/fpsyt.2019.00535 PMC6676343 31404318

[CR41] Halladay, A.K., Bishop, S., Constantino, J.N., Daniels, A.M., Koenig, K., Palmer, K., … Szatmari, P. (2015). Sex and gender differences in autism spectrum disorder: Summarizing evidence gaps and identifying emerging areas of priority. *Molecular Autism, 6*. 10.1186/s13229-015-0019-y 10.1186/s13229-015-0019-y PMC4465158 26075049

[CR42] Haney, J. L. (2016). Autism, females, and the DSM-5: Gender bias in autism diagnosis. *Social Work in Mental Health,**14*(4), 396–407. 10.1080/15332985.2015.1031858

[CR43] Hansen, S. N., Schendel, D. E., & Parner, E. T. (2015). Explaining the increase in the prevalence of autism spectrum disorders. *JAMA Pediatrics,**169*(1), 56. 10.1001/jamapediatrics.2014.1893 25365033 10.1001/jamapediatrics.2014.1893

[CR44] Harkins, C. M., Handen, B. L., & Mazurek, M. O. (2022). The impact of the comorbidity of ASD and ADHD on social impairment. *Journal of Autism and Developmental Disorders,**52*(6), 2512–2522. 10.1007/s10803-021-05150-1 34181141 10.1007/s10803-021-05150-1

[CR45] Harrop, C., Jones, D., Zheng, S., Nowell, S., Schultz, R., & Parish-Morris, J. (2019). Visual attention to faces in children with autism spectrum disorder: Are there sex differences? *Molecular Autism*, *10*(1). 10.1186/s13229-019-0276-2 10.1186/s13229-019-0276-2 PMC6599259 31297179

[CR46] Harrop, C., Gulsrud, A., & Kasari, C. (2015). Does gender moderate core deficits in ASD? An investigation into restricted and repetitive behaviors in girls and boys with ASD. *Journal of Autism and Developmental Disorders,**45*(11), 3644–3655. 10.1007/s10803-015-2511-9 26111739 10.1007/s10803-015-2511-9 PMC4609592

[CR47] Harrop, C., Jones, D. R., Sasson, N. J., Zheng, S., Nowell, S. W., & Parish-Morris, J. (2020). Social and object attention is influenced by biological sex and toy gender-congruence in children with and without autism. *Autism Research,**13*(5), 763–776. 10.1002/aur.2245 31799774 10.1002/aur.2245 PMC8034588

[CR48] Harrop, C., Jones, D., Zheng, S., Nowell, S., Boyd, B. A., & Sasson, N. (2018a). Circumscribed interests and attention in autism: The role of biological sex. *Journal of Autism and Developmental Disorders,**48*(10), 3449–3459. 10.1007/s10803-018-3612-z 29777472 10.1007/s10803-018-3612-z PMC6267775

[CR49] Harrop, C., Jones, D., Zheng, S., Nowell, S. W., Boyd, B. A., & Sasson, N. (2018b). Sex differences in social attention in autism spectrum disorder. *Autism Research,**11*(9), 1264–1275. 10.1002/aur.1997 30403327 10.1002/aur.1997 PMC7468514

[CR50] Harrop, C., Shire, S., Gulsrud, A., Chang, Y.-C., Ishijima, E., Lawton, K., & Kasari, C. (2015). Does gender influence core deficits in ASD? An investigation into social-communication and play of girls and boys with ASD. *Journal of Autism and Developmental Disorders,**45*(3), 766–777. 10.1007/s10803-014-2234-3 25217088 10.1007/s10803-014-2234-3

[CR51] Harrop, C., Green, J., Hudry, K., & PACT Consortium. (2017). Play complexity and toy engagement in preschoolers with autism spectrum disorder: Do girls and boys differ? *Autism,**21*(1), 37–50. 10.1177/1362361315622410 26936930 10.1177/1362361315622410

[CR52] Head, A. M., Mcgillivray, J. A., & Stokes, M. A. (2014). Gender differences in emotionality and sociability in children with autism spectrum disorders. *Molecular Autism,**5*(1), 19. 10.1186/2040-2392-5-19 24576331 10.1186/2040-2392-5-19 PMC3945617

[CR53] Hiller, R. M., Young, R. L., & Weber, N. (2014). Sex differences in autism spectrum disorder based on DSM-5 criteria: Evidence from clinician and teacher reporting. *Journal of Abnormal Child Psychology,**42*(8), 1381–1393. 10.1007/s10802-014-9881-x 24882502 10.1007/s10802-014-9881-x

[CR54] Hiller, R. M., Young, R. L., & Weber, N. (2016). Sex differences in pre-diagnosis concerns for children later diagnosed with autism spectrum disorder. *Autism,**20*(1), 75–84. 10.1177/1362361314568899 25717130 10.1177/1362361314568899

[CR55] Hull, L., Levy, L., Lai, M.C., Petrides, K.V., Baron-Cohen, S., Allison, C., Smith, P., & Mandy, W. (2021). Is social camouflaging associated with anxiety and depression in autistic adults? *Molecular Autism*, *12*(1). 10.1186/s13229-021-00421-1 10.1186/s13229-021-00421-1 PMC7885456 33593423

[CR56] Hull, L., Lai, M. C., Baron-Cohen, S., Allison, C., Smith, P., Petrides, K. V., & Mandy, W. (2020). Gender differences in self-reported camouflaging in autistic and non-autistic adults. *Autism,**24*(2), 352–363. 10.1177/1362361319864804 31319684 10.1177/1362361319864804

[CR57] Hull, L., Mandy, W., Lai, M. C., Baron-Cohen, S., Allison, C., Smith, P., & Petrides, K. V. (2019). Development and validation of the Camouflaging Autistic Traits Questionnaire (CAT-Q). *Journal of Autism and Developmental Disorders,**49*(3), 819–833. 10.1007/s10803-018-3792-6 30361940 10.1007/s10803-018-3792-6 PMC6394586

[CR58] Hull, L., Petrides, K. V., Allison, C., Smith, P., Baron-Cohen, S., Lai, M. C., & Mandy, W. (2017). “Putting on my best normal”: Social camouflaging in adults with autism spectrum conditions. *Journal of Autism and Developmental Disorders,**47*(8), 2519–2534. 10.1007/s10803-017-3166-5 28527095 10.1007/s10803-017-3166-5 PMC5509825

[CR59] Hull, L., Petrides, K. V., & Mandy, W. (2020). The female autism phenotype and camouflaging: A narrative review. *Review Journal of Autism and Developmental Disorders,**7*(4), 306–317. 10.1007/s40489-020-00197-9

[CR60] Hull, L., Petrides, K. V., & Mandy, W. (2021). Cognitive predictors of self-reported camouflaging in autistic adolescents. *Journal of Autism and Developmental Disorders,**50*(7), 2454–2465. 10.1007/s10803-019-03986-9 10.1002/aur.2407 33047869

[CR61] Ioannidis, J. P. A., Patsopoulos, N. A., & Rothstein, H. R. (2008). Reasons or excuses for avoiding meta-analysis in forest plots. *BMJ,**336*(7658), 1413–1415. 10.1136/bmj.a117 18566080 10.1136/bmj.a117 PMC2432114

[CR62] James, D., Lam, V. T., Jo, B., & Fung, L. K. (2022). Region-specific associations between gamma-aminobutyric acid A receptor binding and cortical thickness in high-functioning autistic adults. *Autism Research,**15*(6), 1068–1082. 10.1002/aur.2703 35261207 10.1002/aur.2703 PMC9167258

[CR63] Jedrzejewska, A., & Dewey, J. (2022). Camouflaging in autistic and non-autistic adolescents in the modern context of social media. *Journal of Autism and Developmental Disorders,**52*(2), 630–646. 10.1007/s10803-021-04953-6 33748903 10.1007/s10803-021-04953-6

[CR64] Jorgenson, C., Lewis, T., Rose, C., & Kanne, S. (2020). Social camouflaging in autistic and neurotypical adolescents: A pilot study of differences by sex and diagnosis. *Journal of Autism and Developmental Disorders,**50*(12), 4344–4355. 10.1007/s10803-020-04491-7 32270386 10.1007/s10803-020-04491-7

[CR65] Kauschke, C., Van Der Beek, B., & Kamp-Becker, I. (2016). Narratives of girls and boys with autism spectrum disorders: Gender differences in narrative competence and internal state language. *Journal of Autism and Developmental Disorders,**46*(3), 840–852. 10.1007/s10803-015-2620-5 26438638 10.1007/s10803-015-2620-5

[CR66] Kenny, L., Hattersley, C., Molins, B., Buckley, C., Povey, C., & Pellicano, E. (2016). Which terms should be used to describe autism? Perspectives from the UK autism community. *Autism,**20*(4), 442–462. 10.1177/1362361315588200 26134030 10.1177/1362361315588200

[CR67] Key, A. P., Jones, D., & Corbett, B. A. (2022). Sex differences in automatic emotion regulation in adolescents with autism spectrum disorder. *Autism Research,**15*(4), 712–728. 10.1002/aur.2678 35103402 10.1002/aur.2678 PMC9060299

[CR68] Key, A. P., Yan, Y., Metelko, M., Chang, C., Kang, H., Pilkington, J., & Corbett, B. A. (2022). Greater social competence is associated with higher interpersonal neural synchrony in adolescents with autism. *Frontiers in Human Neuroscience,**15*, 790085. 10.3389/fnhum.2021.790085 35069156 10.3389/fnhum.2021.790085 PMC8770262

[CR69] Kiep, M., & Spek, A. (2017). Executive functioning in men and women with an autism spectrum disorder. *Autism Research,**10*(5), 940–948. 10.1002/aur.1721 27874275 10.1002/aur.1721

[CR70] Kirkovski, M., Enticott, P. G., & Fitzgerald, P. B. (2013). A review of the role of female gender in autism spectrum disorders. *Journal of Autism and Developmental Disorders,**43*(11), 2584–2603. 10.1007/s10803-013-1811-1 23525974 10.1007/s10803-013-1811-1

[CR71] Knutsen, J., Crossman, M., Perrin, J., Shui, A., & Kuhlthau, K. (2019). Sex differences in restricted repetitive behaviors and interests in children with autism spectrum disorder: An Autism Treatment Network study. *Autism,**23*(4), 858–868. 10.1177/1362361318786490 30047281 10.1177/1362361318786490 PMC6348057

[CR72] Ko, J. A., Schuck, R. K., Jimenez-Muñoz, M., Penner-Baiden, K. M., & Vernon, T. W. (2022). Brief report: Sex/gender differences in adolescents with autism: socialization profiles and response to social skills intervention. *Journal of Autism and Developmental Disorders, 52*(6), 2812–2818. 10.1007/s10803-021-05127-0 10.1007/s10803-021-05127-0 34114128

[CR73] Kodak, T., & Bergmann, S. (2020). Autism spectrum disorder: Characteristics, associated behaviors, and early intervention. *Pediatric Clinics of North America,**67*(3), 525–535. 10.1016/j.pcl.2020.02.007 32443991 10.1016/j.pcl.2020.02.007

[CR74] Kopp, S., & Gillberg, C. (2011). The Autism Spectrum Screening Questionnaire (ASSQ)-Revised Extended Version (ASSQ-REV): An instrument for better capturing the autism phenotype in girls? A preliminary study involving 191 clinical cases and community controls. *Research in Developmental Disabilities,**32*(6), 2875–2888. 10.1016/j.ridd.2011.05.017 21664105 10.1016/j.ridd.2011.05.017

[CR75] Kreiser, N. L., & White, S. W. (2013). ASD in females: Are we overstating the gender difference in diagnosis? *Clinical Child and Family Psychology Review,**17*(1), 67–84. 10.1007/s10567-013-0148-9 10.1007/s10567-013-0148-9 23836119

[CR76] Kumazaki, H., Muramatsu, T., Kosaka, H., Fujisawa, T. X., Iwata, K., Tomoda, A., Tsuchiya, K., & Mimura, M. (2015). Sex differences in cognitive and symptom profiles in children with high functioning autism spectrum disorders. *Research in Autism Spectrum Disorders,**13–14*, 1–7. 10.1016/j.rasd.2014.12.011

[CR77] Lai, M.C., Baron-Cohen, S., & Buxbaum, J.D. (2015). Understanding autism in the light of sex/gender. *Molecular Autism*, *6*(24). 10.1186/s13229-015-0021-4 10.1186/s13229-015-0021-4 PMC4429357 25973161

[CR78] Lai, M. C., Lombardo, M. V., & Baron-Cohen, S. (2014). Autism. *The Lancet,**383*(9920), 896–910. 10.1016/s0140-6736(13)61539-1 10.1016/S0140-6736(13)61539-1 24074734

[CR79] Lai, M. C., Lombardo, M. V., Ruigrok, A. N., Chakrabarti, B., Auyeung, B., Szatmari, P., Happé, F., & Baron-Cohen, S. (2017). Quantifying and exploring camouflaging in men and women with autism. *Autism,**21*(6), 690–702. 10.1177/1362361316671012 27899710 10.1177/1362361316671012 PMC5536256

[CR80] Lam, K. S., & Aman, M. G. (2007). The Repetitive Behavior Scale-Revised: Independent validation in individuals with autism spectrum disorders. *Journal of Autism and Developmental Disorders,**37*(5), 855–866. 10.1007/s10803-006-0213-z 17048092 10.1007/s10803-006-0213-z

[CR81] Lawrence, K. E., Hernandez, L. M., Fuster, E., Padgaonkar, N. T., Patterson, G., Jung, J., Okada, N. J., Lowe, J. K., Hoekstra, J. N., Jack, A., Aylward, E., Gaab, N., Van Horn, J. D., Bernier, R. A., McPartland, J. C., Webb, S. J., Pelphrey, K. A., Green, S. A., Bookheimer, S. Y., Dapretto, M. (2022). Impact of autism genetic risk on brain connectivity: A mechanism for the female protective effect. *Brain: A Journal of Neurology, 145*(1), 378–387. 10.1093/brain/awab204 10.1093/brain/awab204 PMC8967090 34050743

[CR82] Lee, J. K., Andrews, D. S., Ozturk, A., Solomon, M., Rogers, S., Amaral, D. G., & Nordahl, C. W. (2022). Altered development of amygdala-connected brain regions in males and females with autism. *The Journal of Neuroscience,**42*(31), 6145–6155. 10.1523/jneurosci.0053-22.2022 35760533 10.1523/JNEUROSCI.0053-22.2022 PMC9351637

[CR83] Lehnhardt, F.-G., Falter, C. M., Gawronski, A., Pfeiffer, K., Tepest, R., Franklin, J., & Vogeley, K. (2016). Sex-related cognitive profile in autism spectrum disorders diagnosed late in life: Implications for the female autistic phenotype. *Journal of Autism and Developmental Disorders,**46*(1), 139–154. 10.1007/s10803-015-2558-7 26319250 10.1007/s10803-015-2558-7

[CR84] Libster, N., Knox, A., Engin, S., Geschwind, D., Parish-Morris, J., & Kasari, C. (2022). Personal victimization experiences of autistic and non-autistic children. *Molecular Autism, 13*(1), 51–54. 10.1186/s13229-022-00531-4 10.1186/s13229-022-00531-4 PMC9790117 36566252

[CR85] Livingston, L. A., Shah, P., & Happé, F. (2019). Compensatory strategies below the behavioural surface in autism: A qualitative study. *The Lancet. Psychiatry,**6*(9), 766–777. 10.1016/S2215-0366(19)30224-X 31350208 10.1016/S2215-0366(19)30224-X PMC6706698

[CR86] Loomes, R., Hull, L., & Mandy, W. P. L. (2017). What is the male-to-female ratio in autism spectrum disorder? A systematic review and meta-analysis. *Journal of the American Academy of Child & Adolescent Psychiatry,**56*(6), 466–474. 10.1016/j.jaac.2017.03.013 28545751 10.1016/j.jaac.2017.03.013

[CR87] Lord, C., Luyster, R., Gotham, K., & Guthrie, W. (2012). *Autism diagnostic observation schedule, 2nd edition (ADOS-2) Manual (Part II): Toddler module.* Western Psychological Services.

[CR88] Mandic-Maravic, V., Pejovic-Milovancevic, M., Mitkovic-Voncina, M., Kostic, M., Aleksic-Hil, O., Radosavljev-Kircanski, J., Mincic, T., & Lecic-Tosevski, D. (2015). Sex differences in autism spectrum disorders: Does sex moderate the pathway from clinical symptoms to adaptive behavior? *Scientific Reports,**5*(1), 10418. 10.1038/srep10418 25988942 10.1038/srep10418 PMC4437371

[CR89] Mandy, W., Chilvers, R., Chowdhury, U., Salter, G., Seigal, A., & Skuse, D. (2012). Sex differences in autism spectrum disorder: Evidence from a large sample of children and adolescents. *Journal of Autism and Developmental Disorders,**42*(7), 1304–1313. 10.1007/s10803-011-1356-0 21947663 10.1007/s10803-011-1356-0

[CR90] Mattila, M. L., Kielinen, M., Linna, S. L., Jussila, K., Ebeling, H., Bloigu, R., Joseph, R. M., & Moilanen, I. (2011). Autism spectrum disorders according to DSM-IV-TR and comparison with DSM-5 draft criteria: An epidemiological study. *Journal of the American Academy of Child and Adolescent Psychiatry,**50*(6), 583-592.e11. 10.1016/j.jaac.2011.04.001 21621142 10.1016/j.jaac.2011.04.001

[CR91] May, T., Cornish, K., & Rinehart, N. (2014). Does gender matter? A one year follow-up of autistic, attention and anxiety symptoms in high-functioning children with autism spectrum disorder. *Journal of Autism and Developmental Disorders,**44*(5), 1077–1086. 10.1007/s10803-013-1964-y 24105364 10.1007/s10803-013-1964-y

[CR92] McFayden, T. C., Albright, J., Muskett, A. E., & Scarpa, A. (2019). Brief report: Sex differences in ASD diagnosis—A brief report on restricted interests and repetitive behaviors. *Journal of Autism and Developmental Disorders,**49*(4), 1693–1699. 10.1007/s10803-018-3838-9 30488150 10.1007/s10803-018-3838-9

[CR93] McPartland, J., Law, K., & Dawson, G. (2016). Autism spectrum disorder. in H. Friedman (Ed.), *Encyclopaedia of Mental Health* (2ed., Vol. 2, pp. 124–130). Elsevier. 10.1016/B978-0-12-397045-9.00230-5

[CR94] McQuaid, G. A., Lee, N. R., & Wallace, G. L. (2022). Camouflaging in autism spectrum disorder: Examining the roles of sex, gender identity, and diagnostic timing. *Autism,**26*(2), 552–559. 10.1177/13623613211042131 34420418 10.1177/13623613211042131

[CR95] Milne, S., Campbell, L., & Cottier, C. (2019). Accurate assessment of functional abilities in pre-schoolers for diagnostic and funding purposes: A comparison of the Vineland-3 and the PEDI-CAT. *Australian Occupational Therapy Journal,**67*, 31–38. 10.1111/1440-1630.12619 31657029 10.1111/1440-1630.12619

[CR96] Milner, V., Mandy, W., Happé, F., & Colvert, E. (2022). Sex differences in predictors and outcomes of camouflaging: Comparing diagnosed autistic, high autistic trait and low autistic trait young adults. *Autism, 27*(2), 402–414. 10.1177/13623613221098240 10.1177/13623613221098240 PMC9902997 35652328

[CR97] Mullen, E. M. (1995). *Mullen Scales of Early Learning*. Pearson (AGS).

[CR98] Mussey, J. L., Ginn, N. C., & Klinger, L. G. (2017). Are males and females with autism spectrum disorder more similar than we thought? *Autism,**21*(6), 733–737. 10.1177/1362361316682621 28749236 10.1177/1362361316682621

[CR99] Nasca, B. C., Lopata, C., Donnelly, J. P., Rodgers, J. D., & Thomeer, M. L. (2020). Sex differences in externalizing and internalizing symptoms of children with ASD. *Journal of Autism and Developmental Disorders,**50*(9), 3245–3252. 10.1007/s10803-019-04132-8 31278524 10.1007/s10803-019-04132-8

[CR100] Neuhaus, E., Lowry, S. J., Santhosh, M., Kresse, A., Edwards, L. A., Keller, J., Libsack, E. J., Kang, V. Y., Naples, A., Jack, A., Jeste, S., McPartland, J. C., Aylward, E., Bernier, R., Bookheimer, S., Dapretto, M., Van Horn, J. D., Pelphrey, K., Webb, S. J., & ACE, G. N. (2021). Resting state EEG in youth with ASD: Age, sex, and relation to phenotype. *Journal of Neurodevelopmental Disorders, 13*(1). 10.1186/s11689-021-09390-1 10.1186/s11689-021-09390-1 PMC8439051 34517813

[CR101] Neuhaus, E., Kang, V. Y., Kresse, A., Corrigan, S., Aylward, E., Bernier, R., Bookheimer, S., Dapretto, M., Jack, A., Jeste, S., McPartland, J. C., Van Horn, J. D., Pelphrey, K., Webb, S. J., & ACE GENDAAR Consortium. (2022). Language and aggressive behaviors in male and female youth with autism spectrum disorder. *Journal of Autism and Developmental Disorders, 52*(1), 454–462. 10.1007/s10803-020-04773-0 10.1007/s10803-020-04773-0 PMC9407024 33682042

[CR102] Nowell, S. W., Watson, L. R., Boyd, B., & Klinger, L. G. (2019). Efficacy study of a social communication and self-regulation intervention for school-age children with autism spectrum disorder: A randomized controlled trial. *Language, Speech, and Hearing Services in Schools,**50*(3), 416–433. 10.1044/2019_LSHSS-18-0093 31287766 10.1044/2019_LSHSS-18-0093 PMC7838961

[CR103] O’Connor, R. A. G., van den Bedem, N., Blijd-Hoogewys, E. M. A., Stockmann, L., & Rieffe, C. (2022). Friendship quality among autistic and non-autistic (pre-) adolescents: Protective or risk factor for mental health? *Autism,**26*(8), 2041–2051. 10.1177/13623613211073448 35068188 10.1177/13623613211073448 PMC9597130

[CR104] Osório, J. M. A., Rodríguez-Herreros, B., Richetin, S., Junod, V., Romascano, D., Pittet, V., Chabane, N., Jequier Gygax, M., & Maillard, A. M. (2021). Sex differences in sensory processing in children with autism spectrum disorder. *Autism Research,**14*(11), 2412–2423. 10.1002/aur.2580 34288517 10.1002/aur.2580 PMC9290069

[CR105] Oswald, T. M., Winter-Messiers, M. A., Gibson, B., Schmidt, A. M., Herr, C. M., & Solomon, M. (2016). Sex differences in internalizing problems during adolescence in autism spectrum disorder. *Journal of Autism and Developmental Disorders,**46*(2), 624–636. 10.1007/s10803-015-2608-1 26438640 10.1007/s10803-015-2608-1

[CR106] Page, M.J., Mckenzie, J.E., Bossuyt, P.M., Boutron, I., Hoffmann, T.C., Mulrow, C.D., Moher, D. (2021). The PRISMA 2020 statement: An updated guideline for reporting systematic reviews. *BMJ*, 71. 10.1136/bmj.n71 10.1136/bmj.n71 PMC8005924 33782057

[CR107] Parish-Morris, J., Liberman, M. Y., Cieri, C., Herrington, J. D., Yerys, B. E., Bateman, L., Donaher, J., Ferguson, E., Pandey, J., & Schultz, R. T. (2017). Linguistic camouflage in girls with autism spectrum disorder. *Molecular Autism*, *8*(1). 10.1186/s13229-017-0164-6 10.1186/s13229-017-0164-6 PMC5622482 29021889

[CR108] Pisula, E., Pudło, M., Słowińska, M., Kawa, R., Strząska, M., Banasiak, A., & Wolańczyk, T. (2017). Behavioral and emotional problems in high-functioning girls and boys with autism spectrum disorders: Parents’ reports and adolescents’ self-reports. *Autism,**21*(6), 738–748. 10.1177/1362361316675119 27899716 10.1177/1362361316675119 PMC5536253

[CR109] Posserud, M., Skretting Solberg, B., Engeland, A., Haavik, J., & Klungsøyr, K. (2021). Male to female ratios in autism spectrum disorders by age, intellectual disability and attention-deficit/hyperactivity disorder. *Acta Psychiatrica Scandinavica,**144*(6), 635–646. 10.1111/acps.13368 34494265 10.1111/acps.13368

[CR110] Postorino, V., Fatta, L. M., De Peppo, L., Giovagnoli, G., Armando, M., Vicari, S., & Mazzone, L. (2015). Longitudinal comparison between male and female preschool children with autism spectrum disorder. *Journal of Autism and Developmental Disorders,**45*(7), 2046–2055. 10.1007/s10803-015-2366-0 25633919 10.1007/s10803-015-2366-0

[CR111] Prosperi, M., Turi, M., Guerrera, S., Napoli, E., Tancredi, R., Igliozzi, R., Apicella, F., Valeri, G., Lattarulo, C., Gemma, A., Santocchi, E., Calderoni, S., Muratori, F., & Vicari, S. (2021). Sex differences in autism spectrum disorder: An investigation on core symptoms and psychiatric comorbidity in preschoolers. *Frontiers in Integrative Neuroscience,**14*, 594082. 10.3389/fnint.2020.594082 33584212 10.3389/fnint.2020.594082 PMC7876072

[CR112] Ratto, A. B., Kenworthy, L., Yerys, B. E., Bascom, J., Wieckowski, A. T., White, S. W., Wallace, G. L., Pugliese, C., Schultz, R. T., Ollendick, T. H., Scarpa, A., Seese, S., Register-Brown, K., Martin, A., & Anthony, L. G. (2018). What about the girls? Sex-based differences in autistic traits and adaptive skills. *Journal of Autism and Developmental Disorders,**48*(5), 1698–1711. 10.1007/s10803-017-3413-9 29204929 10.1007/s10803-017-3413-9 PMC5925757

[CR113] Rea, H. M., Øien, R. A., Shic, F., Webb, S. J., & Ratto, A. B. (2023). Sex differences on the ADOS-2. *Journal of Autism and Developmental Disorders,**53*(7), 2878–2890. 10.1007/s10803-022-05566-3 35451672 10.1007/s10803-022-05566-3 PMC10074828

[CR114] Reinhardt, V. P., Wetherby, A. M., Schatschneider, C., & Lord, C. (2015). Examination of sex differences in a large sample of young children with autism spectrum disorder and typical development. *Journal of Autism and Developmental Disorders,**45*(3), 697–706. 10.1007/s10803-014-2223-6 25189824 10.1007/s10803-014-2223-6 PMC4342305

[CR115] Rivet, T. T., & Matson, J. L. (2011). Review of gender differences in core symptomatology in autism spectrum disorders. *Research in Autism Spectrum Disorders,**5*(3), 957–976. 10.1016/j.rasd.2010.12.003

[CR116] Rodgers, J. D., Lodi-Smith, J., Donnelly, J. P., Lopata, C., Mcdonald, C. A., Thomeer, M. L., Lipinski, A. M., Nasca, B. C., & Booth, A. J. (2019). Brief report: Examination of sex-based differences in ASD symptom severity among high-functioning children with ASD using the SRS-2. *Journal of Autism and Developmental Disorders,**49*(2), 781–787. 10.1007/s10803-018-3733-4 30151783 10.1007/s10803-018-3733-4

[CR117] Ros-Demarize, R., Bradley, C., Kanne, S. M., Warren, Z., Boan, A., Lajonchere, C., Park, J., & Carpenter, L. A. (2020). ASD symptoms in toddlers and preschoolers: An examination of sex differences. *Autism Research,**13*(1), 157–166. 10.1002/aur.2241 31747131 10.1002/aur.2241

[CR118] Ross, A., Grove, R., & McAloon, J. (2022). The relationship between camouflaging and mental health in autistic children and adolescents. *Autism Research, 16*(1), 190–199. 10.1002/aur.2859 10.1002/aur.2859 36416274

[CR119] Rubenstein, E., Wiggins, L. D., & Lee, L. C. (2015). A review of the differences in developmental, psychiatric, and medical endophenotypes between males and females with autism spectrum disorder. *Journal of Developmental and Physical Disabilities,**27*(1), 119–139. 10.1007/s10882-014-9397-x 26146472 10.1007/s10882-014-9397-x PMC4490156

[CR120] Rutter, M., Le Couteur, A., & Lord, C. (2003). Autism diagnostic interview - revised. *Western Psychological Services*.

[CR121] Rutter, M., Bailey, A., & Lord, C. (2003). The Social Communication Questionnaire. *Western Psychological Services*.

[CR122] Rynkiewicz, A., Schuller, B., Marchi, E., Piana, S., Camurri, A., Lassalle, A., & Baron-Cohen, S. (2016). An investigation of the ‘female camouflage effect’ in autism using a computerized ADOS-2 and a test of sex/gender differences. *Molecular Autism,**7*, 10. 10.1186/s13229-016-0073-0 26798446 10.1186/s13229-016-0073-0 PMC4721191

[CR123] Schuck, R. K., Flores, R. E., & Fung, L. K. (2019). Brief report: Sex/gender differences in symptomology and camouflaging in adults with autism spectrum disorder. *Journal of Autism and Developmental Disorders,**49*(6), 2597–2604. 10.1007/s10803-019-03998-y 30945091 10.1007/s10803-019-03998-y PMC6753236

[CR124] Sedgewick, F., Hill, V., & Pellicano, E. (2019). ‘It’s different for girls’: Gender differences in the friendships and conflict of autistic and neurotypical adolescents. *Autism,**23*(5), 1119–1132. 10.1177/1362361318794930 30280923 10.1177/1362361318794930

[CR125] Sedgewick, F., Hill, V., Yates, R., Pickering, L., & Pellicano, E. (2016). Gender differences in the social motivation and friendship experiences of autistic and non-autistic adolescents. *Journal of Autism and Developmental Disorders,**46*(4), 1297–1306. 10.1007/s10803-015-2669-1 26695137 10.1007/s10803-015-2669-1 PMC4786616

[CR126] Song, D., Kim, S. Y., Bong, G., Kim, Y. A., Kim, J. H., Kim, J. -., & Yoo, H. J. (2021). Exploring sex differences in the manifestation of autistic traits in young children. *Research in Autism Spectrum Disorders, 88.*10.1016/j.rasd.2021.101848

[CR127] Song, A., Cola, M., Plate, S., Petrulla, V., Yankowitz, L., Pandey, J., Schultz, R. T., & Parish-Morris, J. (2021). Natural language markers of social phenotype in girls with autism. *Journal of Child Psychology and Psychiatry,**62*(8), 949–960. 10.1111/jcpp.13348 33174202 10.1111/jcpp.13348 PMC9113519

[CR128] Sparrow, S. S., Cicchetti, D., & Balla, D. A. (2005). *Vineland Adaptive Behavior Scales, Second Edition (Vineland-II)* [Database record]. APA PsycTests. 10.1037/t15164-000

[CR129] Sturrock, A., Marsden, A., Adams, C., & Freed, J. (2020). Observational and reported measures of language and pragmatics in young people with autism: A comparison of respondent data and gender profiles. *Journal of Autism and Developmental Disorders,**50*(3), 812–830. 10.1007/s10803-019-04288-3 31758367 10.1007/s10803-019-04288-3 PMC7010622

[CR130] Sturrock, A., Yau, N., Freed, J., & Adams, C. (2020). Speaking the same language? A preliminary investigation, comparing the language and communication skills of females and males with high-functioning autism. *Journal of Autism and Developmental Disorders,**50*(5), 1639–1656. 10.1007/s10803-019-03920-6 30830491 10.1007/s10803-019-03920-6 PMC7211208

[CR131] Supekar, K., de los Angeles, C., Ryali, S., Cao, K., Ma, T., & Menon, V. (2022). Deep learning identifies robust gender differences in functional brain organization and their dissociable links to clinical symptoms in autism. *The British Journal of Psychiatry,**220*(4), 202–209. 10.1192/bjp.2022.13 35164888 10.1192/bjp.2022.13 PMC9376194

[CR132] Tubío-Fungueiriño, M., Cruz, S., Sampaio, A., Carracedo, A., & Fernández-Prieto, M. (2021). Social camouflaging in females with autism spectrum disorder: A systematic review. *Journal of Autism and Developmental Disorders,**51*(7), 2190–2199. 10.1007/s10803-020-04695-x 32926304 10.1007/s10803-020-04695-x

[CR133] Van Wijngaarden-Cremers, P. J., van Eeten, E., Groen, W. B., Van Deurzen, P. A., Oosterling, I. J., & Van der Gaag, R. J. (2014). Gender and age differences in the core triad of impairments in autism spectrum disorders: A systematic review and meta-analysis. *Journal of Autism and Developmental Disorders,**44*(3), 627–635. 10.1007/s10803-013-1913-9 23989936 10.1007/s10803-013-1913-9

[CR134] Waizbard-Bartov, E., Ferrer, E., Heath, B., Rogers, S. J., Nordahl, C. W., Solomon, M., & Amaral, D. G. (2022). Identifying autism symptom severity trajectories across childhood. *Autism Research,**15*(4), 687–701. 10.1002/aur.2674 35084115 10.1002/aur.2674

[CR135] Walsh, M. J. M., Pagni, B., Monahan, L., Delaney, S., Smith, C. J., Baxter, L., & Braden, B. B. (2021). Sex-related neurocircuitry supporting camouflaging in adults with autism: Female protection insights. *Journal of Autism and Developmental Disorders,**51*(1), 156–169. 10.1007/s10803-020-04583-2

[CR136] Wang, S., Deng, H., You, C., Chen, K., Li, J., Tang, C., Ceng, C., Zou, Y., & Zou, X. (2017). Sex differences in diagnosis and clinical phenotypes of Chinese children with autism spectrum disorder. *Neuroscience Bulletin,**33*(2), 153–160. 10.1007/s12264-017-0102-9 28238115 10.1007/s12264-017-0102-9 PMC5360851

[CR137] Wechsler, D. (1999). Wechsler Abbreviated Scale of Intelligence (WASI) [Database record]. *APA PsycTests*. 10.1037/t15170-000

[CR138] Wechsler, D. (2008). *Wechsler Adult Intelligence Scale-Fourth Edition (WAIS-IV) *[Database record]. *APA PsycTests*. 10.1037/t15169-000

[CR139] White, E. I., Wallace, G. L., Bascom, J., Armour, A. C., Register-Brown, K., Popal, H. S., Ratto, A. B., Martin, A., & Kenworthy, L. (2017). Sex differences in parent-reported executive functioning and adaptive behavior in children and young adults with autism spectrum disorder. *Autism Research,**10*(10), 1653–1662. 10.1002/aur.1811 28568910 10.1002/aur.1811 PMC5721669

[CR140] Wiggins, L. D., Rubenstein, E., Windham, G., Barger, B., Croen, L., Dowling, N., Giarelli, E., Levy, S., Moody, E., Soke, G., Fields, V., & Schieve, L. (2021). Evaluation of sex differences in preschool children with and without autism spectrum disorder enrolled in the study to explore early development. *Research in Developmental Disabilities,**112*, 103897. 10.1016/j.ridd.2021.103897 33610079 10.1016/j.ridd.2021.103897 PMC8215620

[CR141] Wilson, C. E., Murphy, C. M., Mcalonan, G., Robertson, D. M., Spain, D., Hayward, H., Woodhouse, E., Deeley, P. Q., Gillan, N., Ohlsen, J. C., Zinkstok, J., Stoencheva, V., Faulkner, J., Yildiran, H., Bell, V., Hammond, N., Craig, M. C., & Murphy, D. G. (2016). Does sex influence the diagnostic evaluation of autism spectrum disorder in adults? *Autism,**20*(7), 808–819. 10.1177/1362361315611381 26802113 10.1177/1362361315611381 PMC5363500

[CR142] Wiskerke, J., Stern, H., & Igelström, K. (2018). Camouflaging of repetitive movements in autistic female and transgender adults [Preprint]. *Neuroscience*. 10.1101/412619

[CR143] Wood-Downie, H., Wong, B., Kovshoff, H., Cortese, S., & Hadwin, J. A. (2021). Research Review: A systematic review and meta-analysis of sex/gender differences in social interaction and communication in autistic and nonautistic children and adolescents. *The Journal of Child Psychology and Psychiatry,**62*(8), 922–936. 10.1111/jcpp.13337 33137209 10.1111/jcpp.13337

[CR144] Wood-Downie, H., Wong, B., Kovshoff, H., Mandy, W., Hull, L., & Hadwin, J. A. (2021). Sex/gender differences in camouflaging in children and adolescents with autism. *Journal of Autism and Developmental Disorders,**51*(4), 1353–1364. 10.1007/s10803-020-04615-z 32691191 10.1007/s10803-020-04615-z PMC7985051

[CR145] Zhang, Y., Qin, B., Wang, L., Chen, J., Cai, J., & Li, T. (2022). Sex differences of language abilities of preschool children with autism spectrum disorder and their anatomical correlation with Broca and Wernicke areas. *Frontiers in Pediatrics,**10*, 762621. 10.3389/fped.2022.762621 35935349 10.3389/fped.2022.762621 PMC9354665

