# Supplementary material for: Is There a Bias Towards Males in the Diagnosis of Autism? A Systematic Review and Meta-Analysis
Source: Neuropsychol Rev. 2024 Jan 29;35(1):153–76. doi: 10.1007/s11065-023-09630-2 (PMC11965184; doi:10.1007/s11065-023-09630-2)
Supplement: Supplementary file 1 — Supplementary file1 (PDF 2384 KB) [file 11065_2023_9630_MOESM1_ESM.pdf]

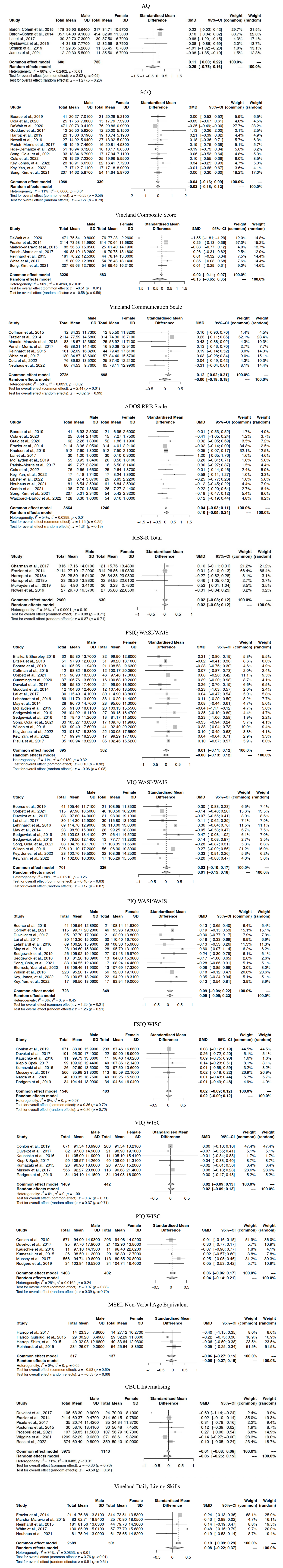
AQ
SCQ
Vineland Composite Score
Vineland Communication Scale
ADOS RRB Scale
RBS-R Total
FSIQ WASI/WAIS
VIQ WASI/WAIS
PIQ WASI/WAIS
FSIQ WISC
VIQ WISC
PIQ WISC
MSEL Non-Verbal Age Equivalent
CBCL Internalising
Vineland Daily Living Skills
