# Supplementary material for: Is There a Bias Towards Males in the Diagnosis of Autism? A Systematic Review and Meta-Analysis
Source: Neuropsychol Rev. 2024 Jan 29;35(1):153–76. doi: 10.1007/s11065-023-09630-2 (PMC11965184; doi:10.1007/s11065-023-09630-2)
Supplement: Supplementary file 2 — Supplementary file2 (DOCX 30 KB) [file 11065_2023_9630_MOESM2_ESM.docx]

**Supplementary Material**

**Table 1**

*Assessment of the quality and risk of bias of the articles addressing phenotypic gender differences, according to the Joanna Briggs Institute (JBI) critical appraisal checklist for analytic cross-sectional studies.*

| **Article** | **Items** | | | | | | | | **Overall** |
| --- | --- | --- | --- | --- | --- | --- | --- | --- | --- |
|  | **1** | **2** | **3** | **4** | **5** | **6** | **7** | **8** |  |
| Baron–Cohen et al., 2014 | Yes | Yes | Yes | No | Yes | Yes | Yes | Yes | 7/8 |
| Baron–Cohen et al., 2015 | Yes | Yes | Yes | Yes | Yes | Yes | Yes | Yes | 8/8 |
| Bitshika & Sharpley., 2019 | Yes | No | Yes | Yes | Yes | No | Yes | Yes | 7/8 |
| Bitsika et al., 2018 | Yes | Yes | Yes | Yes | Yes | No | Yes | Yes | 7/8 |
| Boorse et al., 2019 | Yes | Yes | Yes | Yes | Yes | No | Yes | Yes | 7/8 |
| Charman et al., 2017 | Yes | Yes | Yes | Yes | Yes | Unclear | Yes | N/A | 7/7 |
| Coffman et al., 2015 | Yes | No | Yes | Yes | Yes | Yes | Yes | Yes | 7/8 |
| Cola et al., 2020 | Yes | No | Yes | Yes | Yes | Yes | Yes | Yes | 7/8 |
| Cola et al., 2022 | Yes | Yes | Yes | Yes | Yes | Yes | Yes | Yes | 8/8 |
| Conlon et al., 2019 | Yes | No | Unclear | Unclear | Yes | Yes | Yes | Yes | 5/6 |
| Corbett et al., 2021 | Yes | No | Yes | Yes | Yes | Yes | Yes | Yes | 7/8 |
| Craig et al., 2020 | Yes | Yes | Yes | Yes | Yes | Unclear | Yes | N/A | 7/7 |
| Cummings et al., 2020 | Yes | Unclear | Yes | Yes | Yes | Yes | Yes | Yes | 7/7 |
| DaWalt et al., 2020 | Yes | No | Unclear | Unclear | Yes | Yes | Yes | Yes | 5/6 |
| Duvekot et al., 2017 | Yes | Yes | Yes | Yes | Yes | Yes | Yes | Yes | 8/8 |
| Frazier et al., 2014 | Yes | No | Yes | Yes | Yes | Yes | Yes | Yes | 7/8 |
| Goddard et al., 2014 | Yes | No | Yes | Yes | Yes | Yes | Yes | Yes | 7/8 |
| Harrop, Gulsrud, et al., 2015 | Yes | Unclear | Yes | Yes | Yes | Yes | Yes | Yes | 7/7 |
| Harrop, Shire, et al., 2015 | Yes | No | Yes | Yes | Yes | Yes | Yes | Yes | 7/8 |
| Harrop et al., 2017 | Yes | No | Yes | Yes | Yes | Yes | Yes | Yes | 7/8 |
| Harrop et al., 2018a | Yes | Yes | Yes | Yes | Yes | Yes | Yes | Yes | 8/8 |
| Harrop et al., 2018b | Yes | Yes | Yes | Yes | Yes | Yes | Yes | Yes | 8/8 |
| Harrop et al., 2019 | Yes | No | Yes | Yes | Yes | Yes | Yes | Yes | 7/8 |
| James et al., 2022 | Yes | No | Yes | Yes | Yes | Yes | Yes | Yes | 7/8 |
| Kauschke et al., 2016 | Yes | No | Yes | Yes | Yes | Yes | Yes | Yes | 7/8 |
| Key, Jones, et al., 2022 | Yes | Unclear | Yes | Yes | Yes | Yes | Yes | Yes | 7/7 |
| Key, Yan, et al., 2022 | No | Unclear | Yes | Yes | Yes | Yes | Yes | Yes | 6/7 |
| Kiep & Spek et al., 2017 | Yes | No | Yes | Yes | Yes | Yes | Yes | Yes | 7/8 |
| Knutsen et al., 2019 | Yes | Yes | Yes | Yes | Yes | Yes | Yes | Yes | 8/8 |
| Ko et al., 2022 | Yes | No | Yes | Unclear | Yes | Yes | Yes | Yes | 6/7 |
| Kumazaki et al., 2015 | Yes | Yes | Yes | Yes | Yes | Yes | Yes | Yes | 8/8 |
| Lai et al., 2017 | Yes | No | Yes | Yes | Yes | Yes | Yes | Yes | 7/8 |
| Lawrence et al., 2022 | Yes | Yes | Yes | Yes | Yes | Yes | Yes | Yes | 8/8 |
| Lee et al., 2022 | Yes | No | Yes | Yes | Yes | No | Yes | Yes | 6/8 |
| Lehnhardt et al., 2016 | Yes | Yes | Yes | Yes | Yes | Yes | Yes | Yes | 8/8 |
| Libster et al., 2022 | Yes | Yes | Yes | Yes | Yes | Yes | Yes | Yes | 8/8 |
| Mandic−Maravic et al., 2015 | Yes | Yes | Yes | Yes | Yes | Unclear | Yes | Yes | 7/7 |
| May et al., 2014 | Yes | Yes | Yes | Yes | Yes | Unclear | Yes | N/A | 6/6 |
| McFayden et al., 2019 | Unclear | Yes | Unclear | Yes | Yes | N/A | Yes | Yes | 6/6 |
| Milner et al., 2022 | Yes | Unclear | Yes | Yes | Yes | Yes | Yes | Yes | 7/7 |
| Mussey et al., 2017 | Yes | No | Yes | Yes | Unclear | Unclear | Yes | Yes | 5/6 |
| Nasca et al., 2020 | Yes | Yes | Yes | Yes | Yes | Yes | Yes | Yes | 8/8 |
| Neuhaus et al., 2021 | Yes | Yes | Yes | Yes | Yes | Yes | Yes | Yes | 8/8 |
| Neuhaus et al., 2022 | Yes | Yes | Yes | Yes | Yes | Yes | Yes | Yes | 8/8 |
| Nowell et al., 2019 | Yes | Yes | Yes | Yes | Yes | Unclear | Yes | N/A | 8/8 |
| O`Connor et al., 2022 | Yes | Yes | Yes | Yes | Yes | Yes | Yes | Yes | 8/8 |
| Osório et al., 2021 | Yes | Yes | Yes | Yes | Yes | Yes | Unclear | Yes | 7/7 |
| Parrish–Morris et al., 2017 | Yes | Yes | Yes | Yes | Yes | Yes | Yes | Yes | 8/8 |
| Pisula et al., 2017 | Yes | Yes | Yes | Yes | Yes | Yes | Yes | Yes | 8/8 |
| Postorino et al., 2015 | Yes | Yes | Yes | Yes | Yes | Yes | Yes | Yes | 8/8 |
| Prosperi et al., 2021 | Yes | Unclear | Yes | Yes | Yes | Yes | Yes | Yes | 7/7 |
| Reinhardt et al., 2015 | Yes | Yes | Unclear | Yes | Yes | Yes | Unclear | Yes | 6/6 |
| Rodgers et al., 2019 | Yes | No | Yes | Yes | Unclear | Unclear | Yes | Yes | 5/6 |
| Ros–Demarize et al., 2020 | Yes | No | Yes | Unclear | Yes | Unclear | Yes | Yes | 5/6 |
| Ross et al., 2022 | Yes | No | Unclear | Yes | Unclear | Yes | Yes | Yes | 5/6 |
| Rynkiewicz et al., 2016 | Yes | Yes | Yes | Yes | Yes | Yes | Unclear | Yes | 7/7 |
| Schuck et al., 2019 | Yes | Yes | Yes | Yes | Yes | Unclear | Yes | Yes | 7/7 |
| Sedgewick et al., 2016 | Yes | Yes | Yes | Yes | Yes | N/A | Yes | Yes | 7/7 |
| Sedgewick et al., 2019 | Yes | No | Yes | Yes | Yes | Unclear | Yes | Yes | 6/6 |
| Song, Cola, et al., 2021 | Yes | Yes | Yes | Yes | Yes | N/A | Yes | Yes | 7/7 |
| Song, Kim, et al., 2021 | Yes | Yes | Yes | Yes | Yes | Yes | Yes | Yes | 8/8 |
| Sturrock, Yau, et al., 2020 | Yes | Yes | Yes | Yes | Yes | Yes | Yes | Yes | 8/8 |
| Supekar et al., 2022 | Yes | Yes | Yes | Yes | Yes | Yes | Unclear | Yes | 8/8 |
| Waizbard–Bartov et al., 2022 | Yes | Unclear | Yes | Yes | Yes | Yes | Yes | Yes | 7/7 |
| Wang et al., 2017 | Yes | Unclear | Yes | Yes | Yes | N/A | Yes | Yes | 6/6 |
| White et al., 2017 | Yes | Yes | Yes | Yes | Yes | Yes | Yes | Yes | 8/8 |
| Wiggins et al., 2021 | Yes | Yes | Yes | Yes | Yes | Yes | Yes | Yes | 8/8 |
| Wilson et al., 2016 | Yes | Yes | Unclear | Yes | Yes | No | Unclear | Yes | 5/6 |

*Note*: Answers Yes, No, Unclear or Not/Applicable (N/A).

1. Were the criteria for inclusion in the sample clearly defined?

2. Were the study subjects and the setting described in detail?

3. Was the exposure measured in a valid and reliable way?

4. Were objective, standard criteria used for measurement of the condition?

5. Were confounding factors identified?

6. Were strategies to deal with confounding factors stated?

7. Were the outcomes measured in a valid and reliable way?

8. Was appropriate statistical analysis used?

**Table 2**

*Assessment of the quality and risk of bias of the articles addressing camouflaging in autism, according to the Joanna Briggs Institute (JBI) critical appraisal checklist for analytic cross-sectional studies*

| **Article** | **Items** | | | | | | | | **Overall** |
| --- | --- | --- | --- | --- | --- | --- | --- | --- | --- |
|  | **1** | **2** | **3** | **4** | **5** | **6** | **7** | **8** |  |
| Belcher et al., 2022 | Yes | Unclear | Yes | Yes | Yes | Yes | Yes | Yes | 7/7 |
| Milner et al., 2022 | Yes | Unclear | Yes | Yes | Yes | Yes | Yes | Yes | 7/7 |
| Walsh et al., 2021 | Yes | Unclear | Yes | Yes | Yes | Yes | Yes | Yes | 7/7 |
| Cage & Troxell–Whitman, 2019 | Yes | Yes | Yes | Yes | Yes | Yes | Yes | Yes | 8/8 |
| Cook et al., 2021 | Yes | Yes | Yes | Unclear | Unclear | No | No | No | 3/6 |
| Hull, Lai, et al., 2020 | Yes | Yes | Yes | Yes | Unclear | No | Unclear | Yes | 4/5 |
| Hull, Levi, et al., 2021 | Yes | Unclear | Yes | Yes | Yes | No | Unclear | Yes | 5/6 |
| Hull, Petrides, et al., 2021 | Yes | Yes | Yes | Yes | Unclear | Unclear | No | Yes | 5/6 |
| Jedrzejewska & Dewey, 2022 | Yes | Yes | Yes | Yes | Yes | No | Yes | Yes | 7/8 |
| Jorgenson et al., 2020 | Yes | Yes | Yes | Yes | Yes | Unclear | Unclear | Yes | 6/6 |

*Note:* Answers Yes, No, Unclear or Not/Applicable (N/A).

1. Were the criteria for inclusion in the sample clearly defined?

2. Were the study subjects and the setting described in detail?

3. Was the exposure measured in a valid and reliable way?

4. Were objective, standard criteria used for measurement of the condition?

5. Were confounding factors identified?

6. Were strategies to deal with confounding factors stated?

7. Were the outcomes measured in a valid and reliable way?

8. Was appropriate statistical analysis used?
